# Systematic Comparison of Nanopore and Illumina Sequencing for the Detection of Plant Viruses and Viroids Using Total RNA Sequencing Approach

**DOI:** 10.3389/fmicb.2022.883921

**Published:** 2022-05-11

**Authors:** Anja Pecman, Ian Adams, Ion Gutiérrez-Aguirre, Adrian Fox, Neil Boonham, Maja Ravnikar, Denis Kutnjak

**Affiliations:** ^1^Department of Biotechnology and System Biology, National Institute of Biology, Ljubljana, Slovenia; ^2^Jožef Stefan International Postgraduate School, Ljubljana, Slovenia; ^3^Fera Science Ltd., York, United Kingdom; ^4^Institute for Agri-Food Research and Innovation, Newcastle University, Newcastle upon Tyne, United Kingdom

**Keywords:** high-throughput sequencing, plant virus/viroid detection, comparison, nanopore MinION sequencing, Illumina MiSeq sequencing

## Abstract

High-throughput sequencing (HTS) has become an important tool for plant virus detection and discovery. Nanopore sequencing has been rapidly developing in the recent years and offers new possibilities for fast diagnostic applications of HTS. With this in mind, a study was completed, comparing the most established HTS platform (MiSeq benchtop sequencer—Illumina), with the MinION sequencer (Oxford Nanopore Technologies) for the detection of plant viruses and viroids. Method comparisons were performed on five selected samples, containing two viroids, which were sequenced using nanopore technology for the first time and 11 plant viruses with different genome organizations. For all samples, sequencing libraries for the MiSeq were prepared from ribosomal RNA-depleted total RNA (rRNA-depleted totRNA) and for MinION sequencing, direct RNA sequencing of totRNA was used. Moreover, for one of the samples, which contained five different plant viruses and a viroid, three additional variations of sample preparation for MinION sequencing were also used: direct RNA sequencing of rRNA-depleted totRNA, cDNA-PCR sequencing of totRNA, and cDNA-PCR sequencing of rRNA-depleted totRNA. Whilst direct RNA sequencing of total RNA was the quickest of the tested approaches, it was also the least sensitive: using this approach, we failed to detect only one virus that was present in a sample at an extremely low titer. All other MinION sequencing approaches showed improved performance with outcomes similar to Illumina sequencing, with cDNA-PCR sequencing of rRNA-depleted totRNA showing the best performance amongst tested nanopore MinION sequencing approaches. Moreover, when enough sequencing data were generated, high-quality consensus viral genome sequences could be reconstructed from MinION sequencing data, with high identity to the ones generated from Illumina data. The results of this study implicate that, when an appropriate sample and library preparation are selected, nanopore MinION sequencing could be used for the detection of plant viruses and viroids with similar performance as Illumina sequencing. Taken as a balance of practicality and performance, this suggests that MinION sequencing may be an ideal tool for fast and affordable virus diagnostics.

## Introduction

Globalization of agriculture and international trade facilitate the spread of plant viruses and viroids to new geographic regions with unexpected consequences for food production and natural ecosystems (Jones and Naidu, 2019). To decrease the negative impact of viral diseases on crop production and food safety, rapid and generic plant virus or viroid detection technologies (potentially applicable onsite) are needed. Since the first use of high-throughput sequencing (HTS) for generic detection of plant viruses (Adams et al., 2009; Al Rwahnih et al., 2009; Donaire et al., 2009; Kreuze et al., 2009), a range of HTS platforms were developed and became commonly used for plant virus or viroid detection and discovery. The low error rate and relatively “high throughput” of different instruments of the most widely used platforms, such as Illumina, offer many possibilities for plant virus research (Villamor et al., 2019). However, such “high throughput” may not always be necessary, e.g., when analyzing the single or small number of samples in routine diagnostic laboratories, it increases costs. Often, such samples are outsourced to commercial service providers; however, this increases the turnaround time from a couple of days to several weeks and limits the possibilities for quality control of the full process, which might be crucial in some situations. On the other hand, nanopore sequencing implemented by Oxford Nanopore Technologies offers scalable solutions from small flow cells (up to 2.8 Gb of data per run) accessed using a Flongle adapter, through to the MinION flow cells (up to 50 Gb per run) used here to parallel platforms such as the GridION (up to 250 Gb per run) and PromethION (up to 14 Tb per run). MinION sequencing has the potential benefit that the data can be analyzed in real time (Branton and Deamer, 2019). Compared to the established Illumina sequencing, nanopore sequencing enables long-read sequencing (Van Dijk et al., 2018), which can be an advantage for some applications. However, depending on the specific flow cell used, the error rate can reach up to 15% (Ip et al., 2015; Van Dijk et al., 2018), which can limit the potential applications.

One of the first large-scale applications of the MinION sequencer in virology was for real-time genomic surveillance in the Ebola epidemic in West Africa (Quick et al., 2016). The use of MinION for virus detection and investigation is steadily increasing. In human virology and animal virology, MinION has been used to detect dengue (Yamagishi et al., 2017), Zika (Quick et al., 2017) chikungunya, hepatitis C (Greninger et al., 2015), and porcine reproductive and respiratory syndrome virus (Tan et al., 2019) and is at the moment globally utilized for SARS-CoV-2 genomic surveillance (Meredith et al., 2020).

In plant pathology, MinION has been successfully used for the detection of bacteria, fungi, and viruses using RNA or DNA sequencing (Chalupowicz et al., 2019), plum pox virus and *Candidatus* liberibacter asiaticus in plant tissue and insect samples (Bronzato et al., 2018), and viruses in wheat (Fellers et al., 2019) and cassava (Boykin et al., 2019). In several studies (Filloux et al., 2018; Beddoe et al., 2020; Vazquez-Iglesias et al., 2022), both, MinION sequencing and Illumina sequencing, were used for virus detection; however, systematic comparison between established Illumina sequencing and nanopore sequencing for the detection of a wide array of viruses with different genome types is still lacking.

A previous study, comparing the sequencing of small (s)RNA and total (tot)RNA sequencing using the Illumina platform (Pecman et al., 2017), demonstrated that both approaches can be used for the detection of most of the plant viruses and viroids in a sample, and that totRNA sequencing was a better choice for sequencing novel viruses at low titres. In this report, the focus is on testing the performance of the MinION sequencer for a rapid detection of a wide array of plant viruses or viroids using totRNA sequencing. First, a systematic comparison was made of the fastest or simplest approach for this platform involving direct RNA sequencing of total RNA with an established approach based on sequencing ribosomal RNA-depleted total RNA (rRNA-depleted totRNA) using the MiSeq platform (Illumina). Using the methodology described previously (Pecman et al., 2017) several well-characterized samples containing a broad range of plant viruses and viroids with different genome organizations were included in the comparison.

The main advantage of the direct RNA sequencing approach is a simple and fast library preparation protocol (SQK-RNA002) resulting in long reads without PCR bias (Garalde et al., 2018), but unfortunately, a large amount (500 ng) of RNA is required as the input and the error rate is still relatively high (Wongsurawat et al., 2019). Thus, second, a study was completed using one of the samples, which contained five different plant viruses (including one viral species with two different strains) and one viroid which was analyzed using three other approaches: direct RNA sequencing of rRNA-depleted total RNA, cDNA-PCR sequencing of total RNA, and cDNA-PCR sequencing of rRNA-depleted total RNA.

The results obtained using different nanopore sequencing approaches and the Illumina sequencing were compared in terms of suitability for the detection of plant viruses, using complete datasets and rarefied subsets of data. Not all of the nanopore sequencing approaches performed equally well; however, the results demonstrate that some of them can be confidently used for generic detection of different genome types of plant viruses and viroids, since their performance was comparable to the established Illumina sequencing approach.

## Materials and Methods

### Sample Selection and RNA Isolation

**Figure 1:**
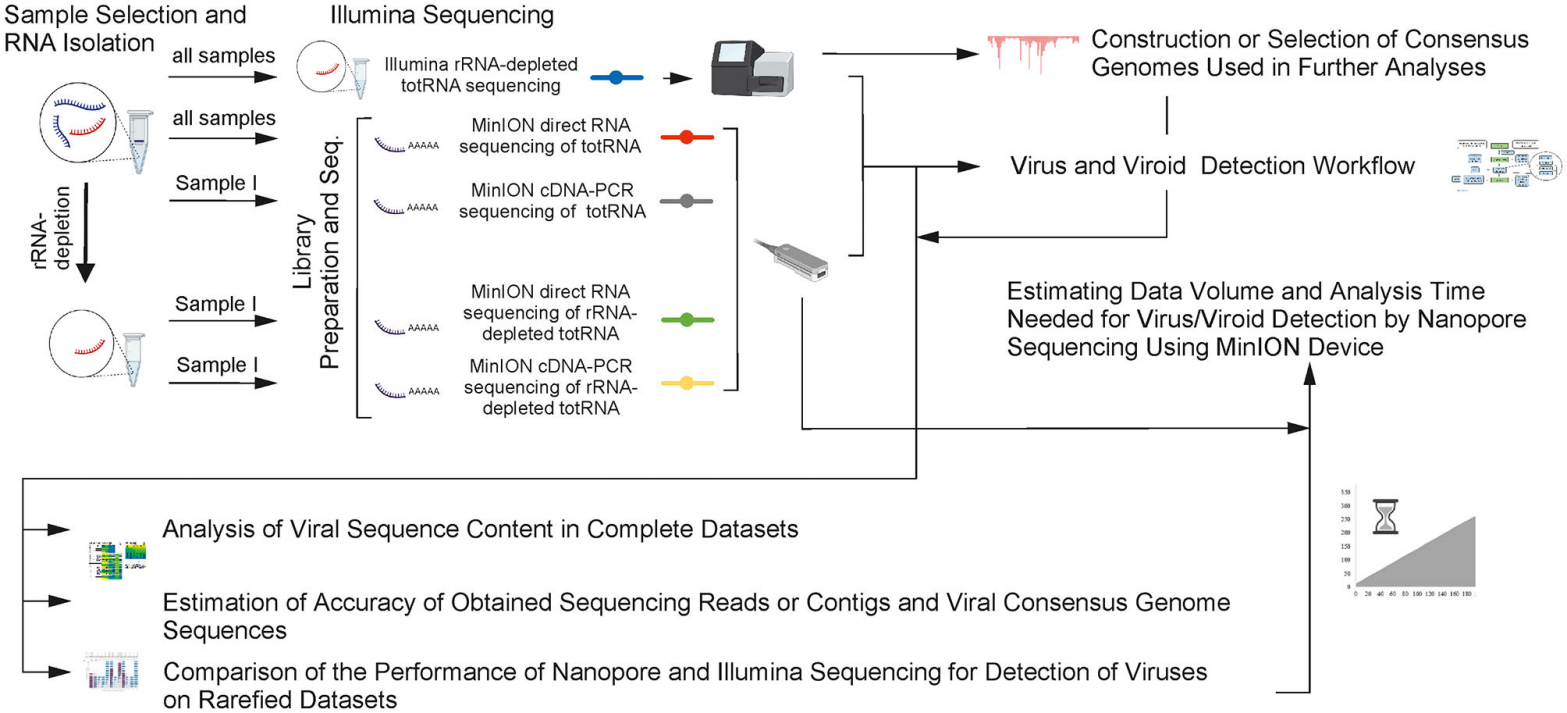
An overview of the conducted study. Listed titles of chapter material and methods are connected with arrows according to the course of work (part of the figure was created with BioRender.com).

To perform an extensive comparison of the methods on a wide array of plant viruses with different genome types, five plant samples were selected, containing different viruses, most of which (samples I, II, and IV) have already been very well characterized for viruses using HTS and targeted detection methods (Pecman et al., 2017). Either infected plant leaf samples (samples I, II, III, and V) or an infected seed sample (sample IV) were used (Table 1; Supplementary Tables S1, S2). Additionally, leaves of healthy tobacco plants were used as a negative control. RNA was isolated (Figure 1) from all leaf samples using the RNeasy Plant Mini Kit (Qiagen) including a DNase step (RNase-Free DNase Set, Qiagen) according to the manufacturer's instructions. From the seed sample (sample IV), RNA was isolated using a combination of CTAB buffer and RNeasy Plant Mini Kit (Qiagen) as described in the study of Adams et al. (2009) with minor modification: incubation with 4 M LiCl was done at 4°C overnight.

**Table 1:** Samples included in the comparison with corresponding results from: HTS (+ virus/viroid detected using pipeline described in section Virus and Viroid Detection Workflow,–virus/viroid not detected using pipeline described in section Virus and Viroid Detection Workflow; NA, not applicable).

**Sample number**	**Host**	**Virus/viroid present (Baltimore classification)**	**Initial detection with HTS sequencing using complete datasets**					**NCBI GenBank accession number**	**NCBI SRA accession number (MinION direct RNA sequencing of totRNA / MinION direct RNA sequencing of rRNA-depleted totRNA / MinION cDNA-PCR sequencing of totRNA / MinION cDNA-PCR sequencing of rRNA-depleted totRNA / Illumina rRNA-depleted totRNA sequencing)**
			**MinION direct RNA sequencing of totRNA**	**MinION direct RNA sequencing of rRNA-depleted totRNA**	**MinION cDNA-PCR sequencing of totRNA**	**MinION cDNA-PCR sequencing of rRNA-depleted totRNA**	**Illumina rRNA-depleted totRNA sequencing**		
I	*Solanum lycopersicum*	TYLCV (ssDNA)	+	+	+	+	+	KY810789	SRR17660996/SRR17660995/SRR17660994/SRR17660993/SRR17319908
		ToCV (ssRNA+)	+	+	+	+	+	KY810786, KY810787	
		PepMV (ssRNA+)	+	+	+	+	+	KF718832.1 (Pep-MV-EU), JX866666.1 (PepMV-CH)	
		ToMV (ssRNA+)	**-**	+	+	+	+	KY810788	
		STV (dsRNA)	+	+	+	+	+	KY810783	
		CLVd (viroid)	+	+	+	+	+	KY810771	
II	*Brassica oleracea*	CaMV (dsDNA-RT)	+	NA	NA	NA	+	KY810770	SRR17660992/NA/NA/NA/SRR17319907
		CCyV1 (ssRNA-)	+	NA	NA	NA	+	KY810772	
III	*Nicotiana tabaccum*	TSWV (ssRNA-)	+	NA	NA	NA	+	OM112200, OM112201, OM112202	SRR17660991/NA/NA/NA/SRR17319906
IV	*Solanum lycopersicum*	TASVd (viroid)	+	NA	NA	NA	+	KY810784	SRR17660990/NA/NA/NA/SRR17319905
V	*Phaseolus vulgaris*	PVeV1 (dsRNA)	+	NA	NA	NA	+	/	SRR17660989/NA/NA/NA/SRR17319904
		PVeV2 (dsRNA)	+	NA	NA	NA	+	OM112199	
		PVeV3 (dsRNA)	+	NA	NA	NA	+	/	

*Virus/viroid names: tomato yellow leaf curl virus (TYLCV, Begomovirus, Geminiviridae), tomato chlorosis virus (ToCV, Crinivirus, Closteroviridae), pepino mosaic virus (PepMV, Potexvirus, Alphaflexiviridae), tomato mosaic virus (ToMV, Tobamovirus, Virgaviridae), southern tomato virus (STV, Amalgavirus, Amalgaviridae), columnea latent viroid (CLVd, Pospiviroid, Pospiviroidae), cauliflower mosaic virus (CaMV, Caulimovirus, Caulimoviridae), cabbage cytorhabdovirus 1 (CCvY1, Cytorhabdovirus, Rhabdoviridae), tomato spotted wilt orthotospovirus (TSWV, Orthotospovirus, Tospoviridae), tomato apical stunt viroid (TASVd, Pospiviroid, Pospiviroidae), and phaseolus vulgaris alphaendornavirus 1, 2, 3 (PVeV1, 2, 3, Alphaendornavirus, Endornaviridae)*.

A part of the RNA extracted from sample I was further processed: ribosomal RNA was depleted from the extract using the RiboMinusTM Plant Kit for RNA-seq (Invitrogen# A1083808), obtaining two versions of sample I: with and without ribosomal RNA depletion (Figure 1, for more details refer to Supplementary Tables S1, S2).

### Library Preparation and Sequencing

The direct RNA sequencing kit (SQK-RNA002) and cDNA-PCR sequencing kit (SQK-PCS108) required polyA+ RNA as input RNA. Therefore, for each sample, polyA tailing of RNA was performed using E. coli poly(A) polymerase (NEB# M0276). The mixture was incubated at 37°C for 15 min. The reaction was stopped by directly proceeding to the clean-up step with Agencourt® AMPure® XP beads (Beckman Coulter) using 1.8 (AMPure® XP beads): 1 (poly(A) tailing mixture) ratio.

The direct RNA sequencing kit (SQK-RNA002, version DRS_9080_v2_revB_22Nov2018, Oxford Nanopore Technologies) was used to prepare sequencing libraries for all the samples included in the study. The cDNA-PCR sequencing kit (SQK-PCS108, version PCS_9035_v108_revH_26Jun2017, Oxford Nanopore Technologies) was additionally used to prepare libraries from sample I and sample I with ribosomal-depleted RNA.

For all but one library, the recommended amount of RNA input was used (Oxford Nanopore Technologies protocols); however, when preparing the library for direct RNA sequencing of rRNA-depleted totRNA (sample I), 278 ng of RNA, instead of 500 ng of RNA, was used (due to the lower extraction yield). Each library was then sequenced on a separate flow cell (R9.5.1) for 46–48 h using MinION device and MinKNOW software (v18.12.6). The reads were base-called using Guppy v3.1.5 and command: rna_r9.4.1_70bps_hac.cfg / dna_r9.4.1_bps450_hac.cfg –device auto –u_substitution false.

### Illumina Sequencing

Sequencing libraries for each sample were prepared using total RNA and the ScriptSeq™ Complete Plant Leaf Kit (production discontinued, Illumina, USA) which included a ribosomal RNA depletion step. The libraries were sequenced on a MiSeq (Illumina, USA) using a 2x300-bp (V3) cartridge.

### Virus and Viroid Detection Workflow

In the first part of the data analysis, the aim was to investigate how well different sequencing approaches compare in terms of virus detection from complete datasets (Figure 1). To achieve this, established in-house virus detection workflows were used and virus presence was reported as follows.

Illumina reads were analyzed using a pipeline in CLC Genomic Workbench (v12, v21) and additional Diamond BLASTX analysis, as described below. Quality control was performed, then, adapters were removed from all reads, and additionally, reads were trimmed by quality (quality limit = 0.05) and length (all reads shorter than 30 nts were discarded). Trimmed reads were mapped to viral RefSeq (NCBI database, updated 19.05. 2019) and *de novo* assembled. Contigs (longer than 100 nts) were mapped to viral RefSeq (NCBI database, updated 19.05.2019) and unmapped contigs were further analyzed by searching for conserved protein domains using Pfam analysis (v32) (refer to Supplementary Tables S3–S6 for details about the used parameters). Additionally, *de novo* assembled contigs were analyzed with Diamond BLASTX (v0.9.22) (Buchfink et al., 2015) against the NCBI nr database (June 2018). Diamond outputs were taxonomically classified and visualized using Megan 6.19.2 (Huson et al., 2007).

To analyse nanopore sequencing data, a similar pipeline was constructed using tools, which enable analysis of long reads. The statistics and quality of MinION reads were checked using the programs NanoQC v0.8.1, NanoStat v1.1.2, and NanoPlot v1.20.1 (De Coster et al., 2018). The read plots generated using the NanoQC v0.8.1 were inspected for each sample individually and were used to determine how to trim them (length of reads, head of reads, and tail of reads) (Supplementary Table S1) using program NanoFilt 2.5.0 (De Coster et al., 2018). The trimmed reads were again quality checked with NanoStat v1.1.2 and then mapped to the viral RefSeq (NCBI database, updated 19.05.2019) using minimap2 (v2.16-r922) and the commands: minimap2 -ax splice -uf -k14 for direct RNA reads and minimap2 -ax map-ont for cDNA-PCR reads. Reads were also analyzed using Diamond BLASTX (v0.9.22) (Buchfink et al., 2015) with the –frameshift 15–range-culling–sensitive command option. All reads were *de novo* assembled by combining different programs using a Pomoxis (https://github.com/nanoporetech/pomoxis) inspired approach: after fast mapping (minimap2) (Li, 2018) and *de novo* assembly (Miniasm) (Li, 2016), two rounds of the contig correction using racon (Vaser et al., 2017) were run. The script together with corresponding parameters is shown in Supplementary Data 1. The assembled contigs were analyzed using BLASTn against the NCBI nt database and visualized with Megan 6.19.2 (Huson et al., 2007). TASVd (sample V) was not detected by mapping direct RNA MinION reads to the viral RefSeq database, so in the next step, more closely related sequence from NCBI GenBank (KY810784) was used as the reference for reads and contig mapping.

### Construction or Selection of Consensus Genomes Used in Further Analyses

To be able to perform comparisons of different sequencing approaches for different viruses or viroids, complete or near complete consensus genomic sequences of viruses in the samples were obtained. Since some of the samples were identical to the ones from a previous study (Pecman et al., 2017), these reference sequences were already available. For phaseolus vulgaris alphaendornavirus 2 (PVeV2, sample V) and tomato spotted wilt orthotospovirus TSWV (sample III), a reference consensus was generated based on the mapping of Illumina reads and contigs, as previously described (Pecman et al., 2017). For pepino mosaic virus (PepMV), two divergent strains (80% nucleotide identity) were detected in the sample I (PepMV-EU and PepMV-Ch2); thus, in this case, the complete genome sequences of KF718832.1 and JX866666.1 were used in subsequent comparisons as described previously (Pecman et al., 2017). For tomato yellow leaf curl virus (TYLCV) (KY810789), tomato chlorosis virus (ToCV) (KY810786), (KY810787), cauliflower mosaic virus (CaMV) (KY810770), and cabbage cytorhabdovirus 1 (CCyV1) (KY810772), the reference sequences described in the study of Pecman et al., (2017) were used in first step, but due to some mismatches after mapping Illumina reads and contigs to those reference sequences, few nucleotides were changed and “new” consensus genome sequences were used for the purpose of the following analysis only (Supplementary Data 2). In the case of sample V, only PVeV2 complete genome was covered by reads by both approaches, and thus, only this endornavirus was included in further analyses.

### Analysis of Viral Sequence Content in Complete Datasets

To calculate the viral sequence content in the complete datasets, trimmed reads for each dataset were mapped to the corresponding reconstructed or selected viral or viroid reference sequences (Section Construction or Selection of Consensus Genomes Used in Further Analyses). For each sample–virus–sequencing type combination, three parameters were reported: the percentage of mapped reads, average depth (the number of times a nucleotide is covered by a sequencing read averaged across the complete reference genome sequence), and fraction of reference covered by reads.

### Estimation of Accuracy of Obtained Sequencing Reads or Contigs and Viral Consensus Genome Sequences

The next step enabled the investigation of (I) average nanopore reads and contig identities (compared to the corresponding reference sequences) and (II) identities of consensus genome sequences generated after mapping the reads to the reference sequence, from now on named “consensus sequence identity”. (I) To determine the average identities of nanopore sequencing reads (proxy of error rate), and the identities of *de novo* assembled contigs (generated from those reads), compared to reference sequences, reads and contigs were mapped to corresponding viral genome reference sequences. Identities were calculated using Minimap2 PAF output (Pairwise mApping Format) (Li, 2018). In this way, the average BLAST-like alignment identity was calculated for each mapping of nanopore sequencing data, either with reads or with contigs.

(II) If sequencing errors in nanopore reads are relatively random and if there is a substantial number of reads covering the reference genome sequence after mapping, the resulting consensus sequence should be “error corrected”. To test this, extracted consensus sequences (or their fragments–if whole genomes were not obtained) derived from mapping nanopore or Illumina reads to reference sequences were aligned with original reference sequences and pairwise identities were calculated using CLC Workbench Genomics v12, v21. For this analysis, consensus sequences (or their fragments if whole genomes were not obtained) were used if they had at least 1x average coverage in the read mapping step.

### Comparison of the Performance of Nanopore and Illumina Sequencing for Detection of Viruses on Rarefied Datasets

To be able to compare the datasets obtained by different sequencing approaches, complete datasets were rarefied to obtain subsamples with comparable numbers of nucleotides. Depending on the original size (Supplementary Tables S1, S2), datasets from different sequencing approaches generated a different number of subsamples. The largest datasets, contained 1,500 million nucleotides, followed by 1,300, 1,100, 900, 700, 500, 300, 200, 100, 50, 30, and 10 millions of nucleotides (Supplementary Table S7). Each set of subsampled reads was randomly generated using CLC Genomic Workbench (v12, v21) for Illumina reads and Seqtk Sample for MinION reads. Subsampling was repeated five times for each of the subsample sizes for each sample.

The rarefied subsets were used for further comparative analysis of different sequencing approaches. The read subsamples were mapped to reconstructed or selected reference sequences. The reads were also *de novo* assembled and the resulting contigs were mapped to the selected reference sequences (for nanopore approaches as described above, for Illumina approach refer to Supplementary Table S3–S5 for details about the used parameters). Finally, the fractions of reference sequences covered by reads or contigs for different subsamples were calculated and visualized as line or bar plots.

Longer reads or contigs mapping to small, circular genomes, could influence mapping efficiency (Visser et al., 2016). All mappings were performed to an artificially constructed viroid sequence, which was made by joining 10 repeated viroid genome sequences. Parameters for mapping contigs from Illumina rRNA-depleted totRNA dataset to viroids were adjusted (for samples I and IV). The length fraction parameter [CLC Genomic Workbench (v12, v21)] was set to 0.5 (50 %) instead of the 0.90 (90 %) used for viruses. Every mapping was individually inspected, and in cases where contigs were longer than the reference sequence, the fraction of reference covered by contig was reported as 100%.

In the case of sample I, in some subsamples, uneven coverage of the two different PepMV strain genome sequences was observed; thus, we performed additional analyses to test whether those observations are the consequence of the presence of two strains of the same virus present in the dataset. For each sequencing approach, one subsample (in which unequal contig coverage was observed for the two viral strains) was chosen (**Figure 4C**, see ^*^). For each of those chosen subsamples, further analyses were implemented: (i) mapping original subsampled reads to the strain better covered by contigs in the original analysis, (ii) *de novo* assembly of the unmapped reads, and (iii) mapping newly assembled contigs to the corresponding reference genome sequence of the other present PepMV strain.

### Estimating Data Volume and Analysis Time Needed for Virus/Viroid Detection by Nanopore Sequencing Using MinION Device

To obtain the proxy of the speed of virus or viroid detection and compare different employed MinION sequencing approaches, the sequencing time needed to obtain 50% of viral or viroid genome covered by generated reads was calculated. For this, a script (get_cumulative_yield_table.py, Supplementary Data 3) was used to calculate the cumulative yields of reads in gigabases for every 10 min of sequencing from MinION basecalling output files (summary.txt, refer to Supplementary Table S8). According to the rarefaction analysis from section Comparison of the Performance of Nanopore and Illumina Sequencing For Detection of Viruses on Rarefied Datasets (the number of nucleotides estimated to cover more than 50% of a reference viral genome) for each virus or sequencing type combination, the time point at which this was reached was calculated according to the cumulative yields of the reads during sequencing.

## Results

### Nanopore Sequencing Using MinION Device Resulted in Comparable Detection of Plant Viruses/Viroids as Illumina Sequencing

**Figure 2:**
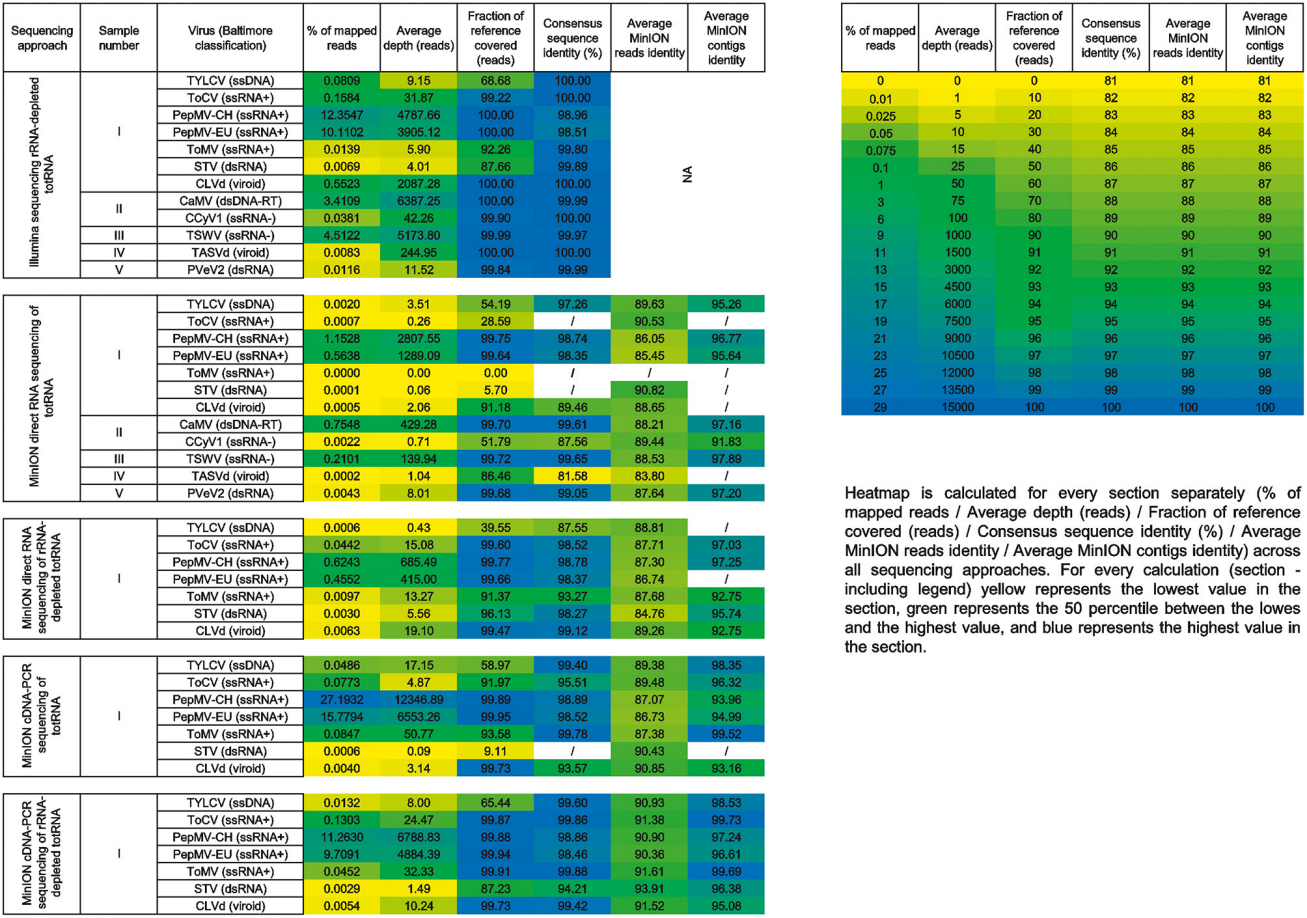
Heatmap of the samples included in the comparison with corresponding results: percentage of mapped reads, average depth (reads), fraction of reference covered (reads), consensus sequence identity (%), and average MinION reads/contigs identity (/ no data; NA not applicable).

**Figure 3:**
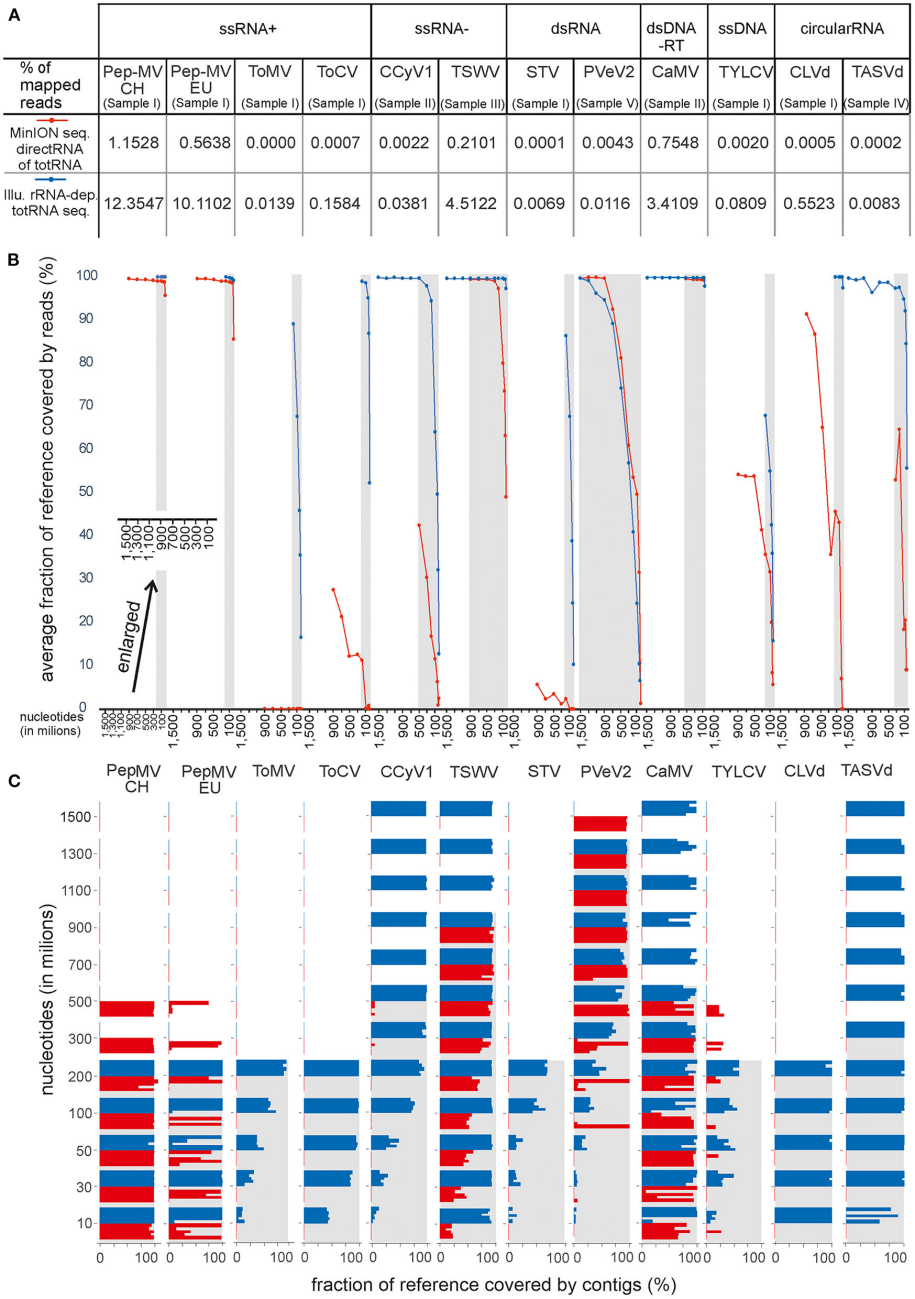
Comparison of MinION direct RNA sequencing of totRNA (represented inred) and Illumina sequencing of rRNA-depleted totRNA (represented in blue) using data size-normalized subsamples. Results for each virus included in the analysis are shown along the x-axis and are grouped according to Baltimore classification. **(A)** Percentage of specific virus reads in trimmed and filtered complete HTS datasets. **(B)** Average fraction of reference covered by reads (%) at different subsample sizes. Dots represent the average value of analysis of 5 replicate subsamples. Different subsample sizes were used (10, 30, 50, 100, 200, 300, 500, 700, 900, 1,100, 1,300, 1,500 million nts–note the enlarged x-axis in the lower left part of panel 3B for a clearer view). **(C)** Fraction of reference covered by contigs (%) at different subsample sizes. Every bar represents the result of analysis for separate replicate subsamples. In **(B,C)**, gray areas designate the range in which the subsamples were available for both approaches compared.

Using the pipeline described above for detection of viral sequences, all viruses except one were detected in the samples employing the MinION direct RNA sequencing approach (Table 1). Using this approach, ToMV, which was present in sample I in an extremely low titer (Figure 2, Figure 3A), was not detected. TASVd (sample IV) was at first not detected by mapping reads to the viral RefSeq database; however, when using a more closely related sequence from NCBI GenBank (KY810784) as the reference, a few reads of this viroid were detected.

When using the three additional MinION sequencing approaches: direct RNA sequencing of rRNA-depleted totRNA, cDNA-PCR sequencing of totRNA, and cDNA-PCR sequencing of rRNA-depleted totRNA, all of the viruses or viroids present in the samples were detected (Figure 2).

### Performance Comparison of MinION Direct RNA Sequencing and Illumina rRNA-Depleted totRNA Sequencing

The analysis of complete datasets showed that Illumina sequencing of rRNA-depleted totRNA resulted in a markedly higher relative fractions of viral reads compared to MinION direct RNA sequencing of totRNA (Figure 3A), which was expected due to the inclusion of the ribosomal RNA depletion step in the Illumina approach. These differences were reflected also when performing additional analyses on rarefied datasets (Figure 3); however, they were dependent on the amount of virus reads present in the original dataset.

As noted in section Nanopore Sequencing Using MinION Device Resulted in Comparable Detection of Plant Viruses/Viroids as Illumina Sequencing, no reads of ToMV were detected by MinION direct RNA sequencing of totRNA even in the complete dataset. Comparisons of rarefied subsamples further showed that MinION direct RNA sequencing performed comparably well to Illumina sequencing in cases in which a high fraction of specific viral reads was present in the samples (Figure 3). For MinION direct RNA datasets, in which virus sequences were present at more than 0.5% (PepMV in sample I and CaMV in sample II), relatively high fractions of corresponding viral genomes were covered by reads and contigs even in the smallest subsamples (Figure 3). Rarefaction analysis also showed very similar performance between the two approaches for PVeV2 (sample V)–in both cases, the fraction of genome covered by reads or contigs dropped correspondingly with the decreased dataset sizes. For TSWV, with 0.2% of the reads mapped to the viral genome, sharp drops in fractions of reference covered by reads or contigs were observed at smaller subsample data sizes for MinION direct RNA sequencing approach. For TYLCV, none of the two approaches enabled reconstruction of the complete genome, and Illumina sequencing approach performed only slightly better considering the two investigated parameters. In this analysis, for the remaining viruses and viroids (ToMV, ToCV, CCyV1, STV, CLVd, and TASVd), the MinION direct RNA sequencing method resulted in lower fraction of the genome covered by reads and contigs when compared to Illumina sequencing. In several cases, *de novo* assembly did not produce any contigs for the corresponding viruses, even for the complete datasets (data not shown).

### Choice of a Suitable MinION Sequencing Approach Can Improve Detection of Plant Viruses and Viroids

Direct RNA sequencing using the MinION enabled detection of most of the plant viruses or viroids that were previously confirmed in the same samples with rRNA-depleted totRNA Illumina sequencing. However, it showed somewhat inferior performance in the systematic comparisons described in a previous section. Thus, a single sample, containing five viruses and one viroid (sample I), was selected and used to further explore the performance of three additional sample preparation or nanopore sequencing library preparation approaches, which included either rRNA depletion, sequencing of PCR-amplified cDNA, or both (Figure 1), to investigate how much of the performance deficit is due to the sample preparation or library preparation method and how much is due to the platform.

**Figure 4:**
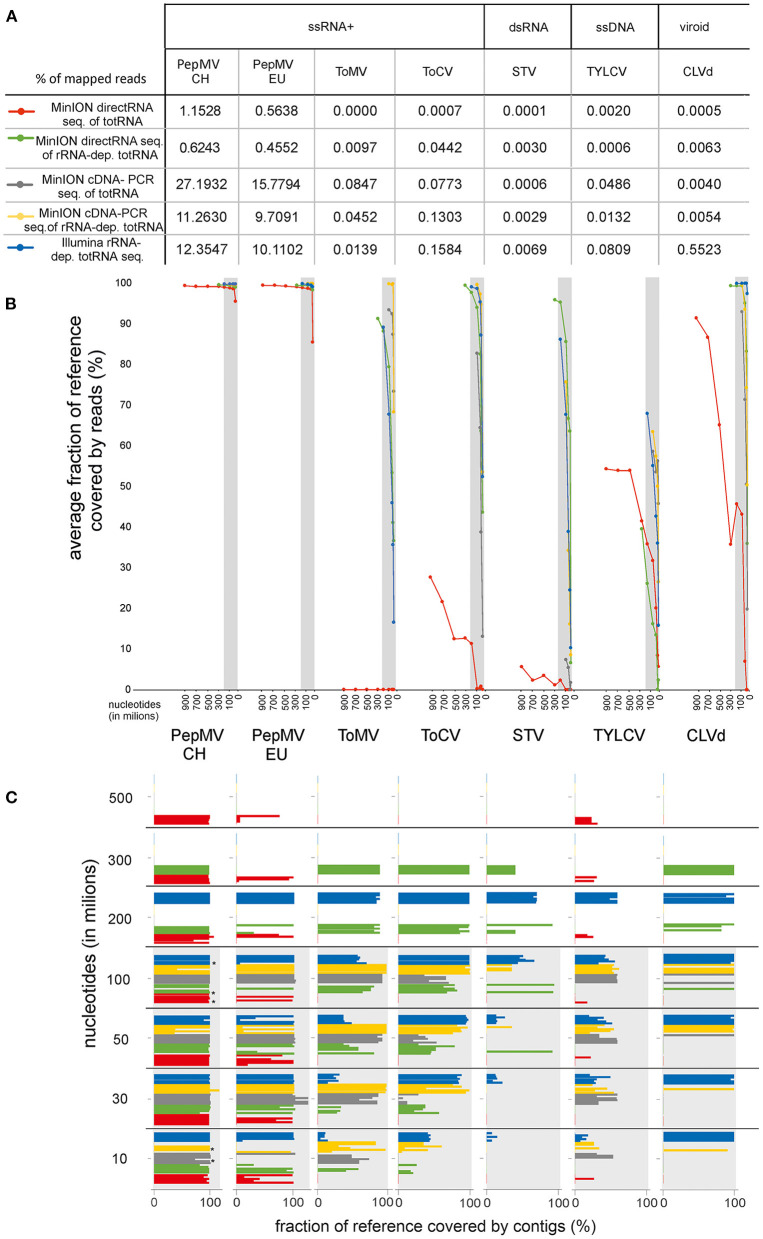
Comparison of MinION sequencing: direct RNA sequencing of totRNA, direct RNA sequencing of rRNA-depleted totRNA, cDNA-PCR sequencing of totRNA, cDNA-PCR sequencing of rRNA-depleted totRNA and Illumina sequencing of rRNA-depleted totRNA using data size-normalized subsamples. Results for each virus included in the analysis are shown along the x-axis and are grouped according to Baltimore classification. **(A)** Percentage of specific virus reads in trimmed and filtered complete HTS datasets. **(B)** Average fraction of reference covered by reads (%) at different subsample sizes. Dots represent the average value of analysis of 5 replicated subsamples. Different subsample sizes were used (10, 30, 50, 100, 200, 300, 500, 700, 900, 1,100, 1,300, 1,500 million nts). **(C)** Fraction of reference covered by contigs (%) at different subsample sizes. Each bar represents the result of analysis for a separate replicate subsample. Bars with * indicate chosen subsample for additional analysis explained in section Comparison of the Performance of Nanopore and Illumina Sequencing for Detection of Viruses on Rarefied Datasets. In **(B,C)**, red represents MinION direct RNA sequencing of totRNA, green represents direct RNA sequencing of rRNA-depleted totRNA, gray represents cDNA-PCR sequencing of totRNA, yellow represents cDNA-PCR sequencing of rRNA-depleted totRNA, and blue represents the results for Illumina sequencing of rRNA-depleted totRNA. Gray areas designate the range in which the subsamples were available for both compared approaches.

Of these approaches, the one which is most comparable to the method used for Illumina sequencing (cDNA-PCR sequencing of rRNA-depleted totRNA) resulted in very similar fractions of viral reads in the sequenced datasets (Figure 4). This approach resulted in lower fractions of specific virus or viroid reads for almost all viruses than observed for both nanopore cDNA-based sequencing approaches or for the Illumina sequencing approach. Compared to MinION direct RNA sequencing without ribosomal RNA depletion, it resulted in an increased fraction of specific viral reads for four out of 7 viruses or viroids, including the detection of one virus (ToMV), which was not detected using direct RNA sequencing alone.

MinION sequencing of cDNA-PCR without ribosomal RNA depletion also resulted in relatively high fractions of specific viral reads. For four out of seven viruses or viroids, the numbers of corresponding reads were even higher than in the rRNA-depleted dataset sequenced by the same method (Figure 4).

Moreover, even though for most viruses, the fractions of viral reads did not increase, when including rRNA depletion to direct RNA sequencing, the rarefaction analysis showed improved performance also in this case, for all but one virus, TYLCV (Figure 4).

For viroids, the Illumina rRNA-depleted totRNA sequencing approach resulted in higher fractions of viroid reads than any of the MinION sequencing approaches (Figures 3A, 4A).

*De novo* assembly of reads from sample I, which contained two strains of PepMV in some subsamples, resulted in an assembly of contigs corresponding only to one strain (Figures 3C, 4C). The effect was observed, when using either Illumina or nanopore sequencing approaches. After the removal of the reads of one or the other PepMV strains and performing *de novo* assembly again (as described in Section Comparison of the Performance of Nanopore and Illumina Sequencing for Detection of Viruses on Rarefied Datasets), the artifact was no longer observed. Additionally, for some subsamples of MinION data, the assembled contigs were longer than the reference genome sequence (Figure 4C). Further investigation (visual inspection of nanopore reads mapping to the corresponding contigs) revealed mistakes or artifacts in some mapped reads, which likely led to artifactual *de novo* assembly of the corresponding contigs.

### Different Nanopore Sequencing Approaches Using MinION Device Result in Different Accuracy of Reads; However, Generated Consensus Sequences Show Relatively High Accuracies in all Cases, When Sequencing Depths Are High Enough

Closer investigation and comparison of average read identities (proxy of sequencing error rate) for different MinION sequencing approaches for sample I revealed the lowest read identities when using direct RNA sequencing of rRNA-depleted totRNA (minimum 84.67%, average 87.43%). The highest average read identities were observed when using cDNA-PCR sequencing of the rRNA-depleted totRNA approach (minimum 90.36%, average 91.5%). For the same approach, also the highest average contig identities were observed (minimum 95.08%, average 99.73%). Even though the inclusion of the ribodepletion step resulted in a slightly decreased mean quality score (Supplementary Table S2), as was already observed in other studies (Wongsurawat et al., 2019), the calculation of average MinION read identity (Figure 2) did not show any marked differences.

As expected, the pairwise identities of consensus viral sequences compared to generated or selected references were higher in the case of Illumina sequencing approach for all viruses or viroids for which a calculation was possible (>99.5% in all cases). For MinION sequencing approaches, pairwise identities of consensus viral sequences compared to generated or selected references were higher than 98%, when average sequencing depth values were 5x or more, except in the case of ToMV (sample I), where, despite the sequencing depth of 13.27, this was 93.27%. Upon visual inspection of the mapping files, we observed that this was a consequence of uneven coverage by reads (the 5‘ of the viral genome was covered by very few reads), which contributed to the final lower average pairwise identity of consensus viral genome sequence.

### Rapid Generation of MinION Data Needed for Detection of Viruses Present in Plants in Moderate Titres

A relatively short time (>30 min) was needed for retrieving sufficient data (covering at least 50% of viral genomic sequences), in cases, in which fractions of specific viral reads in samples were higher than 0.2% (PepMV, CaMV, TSWV; Figures 3, 4; Table 2) using any of the sample preparation approaches. Further, in the case of PVeV2, for which 0.0043% of reads in the sample were mapped to the virus reference sequence, ~2.5 h was needed to retrieve sufficient amount of data. For cases, in which virus or viroid reads were present in samples in very small fractions, a relatively long time was needed to retrieve sufficient amount of data; e.g., for CLVd (0.0005% of reads were mapped to viroid reference sequence), the sequencing should last at least around 12 h to retrieve enough data. Equivalent observations were made for several other viruses or viroid, sequenced within the sample I (Table 2). In some cases, especially when very low fractions of specific viral reads were observed in the samples, retrieving enough data to cover >50% of genome sequence was not achieved even if the sequencing lasted for 46–48 h (Table 2).

**Table 2:** Cumulative yield of nucleotides sequenced in time.

**Sample**	**Virus/viroid**	**Nucleotides**	**Time**	**Nucleotides**	**Time**	**Nucleotides**	**Time**	**Nucleotides**	**Time**
**number**	**present**	**(millions)**	**(minutes)**	**(millions)**	**(minutes)**	**(millions)**	**(minutes)**	**(millions)**	**(minutes)**
		**MinION direct RNA**		**MinION direct RNA sequencing**		**MinION cDNA-PCR**		**MinION cDNA-PCR sequencing**	
		**sequencing of totRNA**		**of rRNA-depleted totRNA**		**sequencing of totRNA**		**of rRNA-depleted totRNA**	
I	TYLCV	500	520	/	/	30	50	50	220
	ToCV	/	/	30	80	50	130	10	20
	PepMV	10	10	10	30	10	10	10	20
	ToMV	/	/	50	140	10	10	10	20
	STV	/	/	30	80	/	/	100	600
	CLVd	700	710	30	80	30	50	10	20
II	CaMV	10	10	NA	NA	NA	NA	NA	NA
	CCyV1	/	/	NA	NA	NA	NA	NA	NA
III	TSWV	20	30	NA	NA	NA	NA	NA	NA
IV	TASVd	200	220	NA	NA	NA	NA	NA	NA
V	PVeV2	200	170	NA	NA	NA	NA	NA	NA

*Data in table represent the time when enough nucleotides would be sequenced to cover at least 50% of virus/viroid reference sequence by mapping reads. / no data: 50% coverage of virus/viroid reference by reads were not achieved. NA, not applicable*.

## Discussion

The systematic comparison of different approaches of nanopore and Illumina sequencing performed in this study demonstrated the effectiveness of nanopore sequencing using the MinION platform (Oxford Nanopore Technologies) for fast and sensitive detection of plant viruses, when the most optimal library preparation approach is used. Besides the ability to detect viruses, the accuracy and time efficiency of the approach were evaluated.

All viruses present in investigated samples, except ToMV, which was present in a sample in extremely low titer (Pecman et al., 2017), were identified using all of the employed approaches. ToMV was not detected using direct RNA sequencing of totRNA with the MinION sequencer (Figure 2). In general, across all datasets, inferior performance of direct RNA sequencing of totRNA using MinION compared to Illumina-based rRNA-depleted totRNA sequencing for detection of plant viruses is evident: between 10.7 and 1,104.6 (PepMV and CLVd, respectively) times, fewer virus reads (average 244.8) were observed in the complete direct RNA MinION datasets compared to the Illumina datasets. Rarefaction analyses showed a similar picture: the fractions of reference viral genomes covered by viral reads or contigs dropped markedly with reduced dataset sizes for direct RNA MinION for viruses, which were present in the original datasets in low amounts (e.g., below 0.2%) (Figure 3). Moreover, in some cases, no contigs were recovered after *de novo* assembly for these viruses (Figure 3C), which indicates that the approach would not be very efficient for detection of (new) viruses present in plants in low titres.

We primarily included direct RNA sequencing of totRNA on MinION in this comparative study due to the speed and straightforward nature of this approach (according to SQK-RNA002, only 115 min is needed for library preparation). However, results of this first comparison showed that such an approach has a reduced performance for detection of plant viruses, compared to the Illumina-based sequencing of rRNA-depleted totRNA, which we take as a ‘golden standard' in this study. This is likely due to both the high error rate of the sequencing itself and the lack of the rRNA depletion step. Thus, in a second part of the study, we performed analysis of a selected sample (containing a diverse assembly of plant viruses) using nanopore sequencing MinION device, but with several improvements, which were shown to improve the performance of nanopore sequencing for virus detection.

Including rRNA depletion prior to the direct RNA sequencing approach using the MinION platform resulted in increased fractions of specific virus reads in most of the cases (Figure 2). The fractions of specific virus reads changed between 0.3 and 63.1 (TYLCV and ToCV, respectively) times, and on average, the fraction increased by 17.8 times for all viruses tested.

The protocol using cDNA-PCR sequencing of totRNA (with no rRNA depletion) also resulted in improvements in the fractions of specific virus reads in the dataset (Figure 2). The fractions of virus reads increased between 6.0 and 110.4 (STV and ToCV, respectively) times compared to direct RNA sequencing of totRNA and on average 33.3 times for all viruses tested. For three viruses (PepMV-EU, PepMV-CH, and ToMV), this approach resulted in a greater proportion (1.5x, 2.2x, and 6.09x, respectively) of virus sequences in the MinION dataset than sequencing rRNA-depleted totRNA using the Illumina platform.

Moreover, incorporating both rRNA depletion and reverse transcription prior to nanopore sequencing using MinION platform (i.e., using cDNA-PCR sequencing of rRNA-depleted totRNA) led to the greatest increases in the observed fractions of specific virus reads. The proportion of virus reads increased between 6.6 and 186.1 (TYLCV and ToCV, respectively) times and on average 43.2 times for all viruses tested compared to direct RNA sequencing. For one virus (ToMV), this approach resulted in a greater proportion (3.2 x) of virus sequences obtained in the respective MinION dataset compared to the dataset generated from rRNA-depleted totRNA using the Illumina platform. Using cDNA-PCR protocol for sequencing rRNA-depleted totRNA also resulted in the highest observed consensus sequence identities, and the comparison of calculated average viral reads identities revealed that this approach resulted in most “accurate” sequencing reads (Figure 2).

As mentioned above, in a number of cases, no contigs of the corresponding viruses were generated from complete (data not shown) or near complete MinION direct RNA datasets (Figures 3, 4). This was also observed for two viruses (TYLCV, PepMV-EU) when direct RNA sequencing of rRNA-depleted RNA was used and one virus (STV) when cDNA-PCR sequencing of totRNA was used. This suggests that detection of such viruses could be missed if only contigs are analyzed, and also, that unknown viruses would be missed if present in samples in similarly low titres. Using the cDNA-PCR protocol for sequencing rRNA-depleted totRNA, contigs were assembled for all viruses in the datasets tested and they showed the highest average contig identity of all of the nanopore sequencing approaches compared (Figures 2, 4).

The performance of the direct RNA sequencing of totRNA using MinION was notably poorer for viroids than viruses. Compared to Illumina sequencing, there was 1104,6x and 41,5x smaller proportion of CLVd and TASVd viroid reads when sequencing totRNA directly using the MinION and for both viroids included in the comparison, only partial genomes were obtained. When using either cDNA-PCR sequencing of rRNA-depleted RNA or direct RNA sequencing of rRNA-depleted totRNA, complete viroid genomes were recovered (Figure 4). Inferior performance of the direct sequencing of totRNA for the detection of viroids is most likely a consequence of the circular genome which could not be polyadenylated in the first steps of the protocol. Only damaged or intermediate replication forms of viroids could be polyadenylated. Moreover, it is possible that the nanopore sequencing could be adversely affected by the secondary structure of viroid RNA (Flores et al., 2014), and this may be partly overcome by the larger amounts of target present in rRNA-depleted totRNA. This study is the first using nanopore sequencing for viroids, and further improvements in template preparation, such as fragmentation of the input RNA, could be envisaged in this case.

Regardless of the approach used (Illumina or all of the tested template preparation methods using the MinION device), we observed artifacts in an assembly of PepMV genomes. PepMV was present in sample I in a mixed infection of two strains. In several cases, the genome of one of the two strains was not assembled in the process. After subtracting the reads of one of the strains and repeating the *de novo* assembly, the artifact was no longer observed. This suggests that special attention should be applied to analysis pipelines to resolve observed assembly artifacts to ensure detection of multiple strains of the same virus.

Different types of input material could possibly also affect the quality and the amount of the generated sequencing data. Most of the samples used in this study were frozen leaves; however, also, fresh leaves and the dry seed samples were used in some cases. Although in our study, the sample size for different input materials is not large enough to draw any conclusions, we observed the highest amount of generated data when using fresh leaf material (Supplementary Table S1). More tests would be needed to study the impact of the input material on the sequencing results and likely specific adaptations of extraction and sample preparation procedures could be implemented to ascertain optimal results for different sample types.

One of the main advantages of the nanopore sequencing is the speed with which sequence data are generated (Bronzato et al., 2018; Fellers et al., 2019). In this study, the libraries for MinION sequencing were prepared and applied to the flow cell within 1 day. Since the data could be analyzed in real time, estimations of the time needed to generate sequences, which would cover 50% or more of a specific viral genome using read mapping were made. The time in which these thresholds were achieved depended on the amount of virus reads present in the complete datasets (a proxy of viral titer). Our estimates suggest the 50% threshold would be achieved in 10–30 min for PepMV using any of the approaches, or on the other end of the spectrum in 11 h and 50 min for CLVd (using MinION direct RNA sequencing of totRNA). This suggests that a diagnostic workflow could be established with <1 day to perform RNA extraction and library preparation, followed by an overnight run on the MinION device and bioinformatic analysis of the following day.

The speed and accessibility of the methodology has led to exploration of the technique as an in-field diagnostic tool (Boykin et al., 2019). Though possible for the detection of high-titer viruses using rapid sample preparation and simplified analysis, this would currently rely on transferring several items of laboratory equipment to the site where testing is being performed and would also need the end-users to be skilled in molecular biology protocols. For diagnostic applications where potentially low-titer infections are likely, the method would require significantly more time to run the flow cell and analyse the data, making it less practical for in-field use. Furthermore, adoption as a field test is dependent on the time criticality of the actions taken based on the outcome of the test and how these will be improved if the results are generated more quickly. The practical benefits described do make the approach suited to routine diagnostic laboratories, where the larger sequencers may be too expensive and impractical to run, leading laboratories to rely on outsourcing to HTS providers. The speed and scalability of MinION sequencing make it well suited to smaller numbers of samples in diagnostic laboratories and, in particular, where rapid turnaround of results is needed especially, given the results are approaching the quality generated by Illumina sequencing.

One of the challenges for introducing this method into routine laboratory use is the constant and rapid development, introduction and withdrawal of flow cells, kits, protocols, and bioinformatic tools by Oxford Nanopore Technologies. This leads to uncertainty with incorporating nanopore into routine testing protocols, in particular those run within a quality certification scheme (e.g., ISO17025). To overcome this obstacle, the use of internal negative control (healthy plant) and in particular a standardized positive control suited for the entire workflow is needed. For example, *Phaseolus vulgaris*, cv. Black Turtle infected with endornaviruses has been used as a positive control in other studies (Kesanakurti et al., 2016) and was successfully sequenced in this study (Sample V).

Finally, when comparing the costs for sample, library preparation, and sequencing per sample using either MiSeq (Illumina) or MinION flow cell (Oxford Nanopore Technologies), comparable prices can be estimated if using multiplexing of samples. In the case of MiSeq (Illumina) sequencing, the estimated cost is 189 €/sample if sequencing 24 samples in the same run (Vazquez-Iglesias et al., 2022). On the other hand, the estimated cost for barcoding and sequencing 12 or 24 samples on one MinION flow cell using cDNA-PCR barcoding kit and including ribodepletion step is 215 €/sample or 170 €/sample, respectively. Due to the increasing throughput of the MinION flow cells, both multiplexing options should now provide enough data for reliable detection of most of the viruses present in the tested samples. More detailed calculation is described in Supplementary Data 4.

To conclude, the results of this study indicate that, when appropriate library preparation and sequencing protocols are selected, nanopore sequencing using the MinION device gave equivalent detection of a range of viruses and viroids than a commonly used Illumina sequencing approach. In this regard, the performance of MinION direct RNA sequencing of totRNA was lower than Illumina sequencing, but improved significantly when rRNA depletion was incorporated or when cDNA amplification or both were incorporated. Whilst these slowed down the sample preparation, they facilitated detection of the lowest titer virus infection included in the study.

## Data Availability Statement

The datasets presented in this study can be found in online repositories. The names of the repository/repositories and accession number(s) can be found in the article/Supplementary Material.

## Author Contributions

AP, DK, MR, and NB designed the experiment. AP performed laboratory part of the experiment and analyzed the data with the assistance of IA and DK and wrote the draft of the manuscript. All authors significantly contributed with reviewing and editing the manuscript.

## Funding

The study was financially supported by Slovenian Research Agency thought project NanoPhyto (L7-2632), core financing (P4-0165, P4-0407), and young researcher grant (AP), Euphresco (VIRFAST) project and by COST Action CA15223, thought STSM (short-term scientific mission).

## Conflict of Interest

IA and AF are employed by Fera Science Ltd. The remaining authors declare that the research was conducted in the absence of any commercial or financial relationships that could be construed as a potential conflict of interest.

## Publisher's Note

All claims expressed in this article are solely those of the authors and do not necessarily represent those of their affiliated organizations, or those of the publisher, the editors and the reviewers. Any product that may be evaluated in this article, or claim that may be made by its manufacturer, is not guaranteed or endorsed by the publisher.
