# Supplementary material for: Systematic Comparison of Nanopore and Illumina Sequencing for the Detection of Plant Viruses and Viroids Using Total RNA Sequencing Approach
Source: Front Microbiol. 2022 May 11;13:883921. doi: 10.3389/fmicb.2022.883921 (PMC9131090; doi:10.3389/fmicb.2022.883921)
Supplement: Supplementary file 1 [file Data_Sheet_1.docx]

Supplementary Material

# Supplementary Data

Supplementary data 1: script with programs and used parameters for *de novo* assembly of MinION reads applied in this study

minimap2 -x ava-ont sample.fastq sample.fastq > sample.paf

miniasm -f sample.fastq sample.paf -c 1 -s 50 -e 1 -n 1 -I 0 -F 0 -1 -2 > sample.gfa

awk '$1 ~/S/ {print ">"$2"\n"$3}' sample.gfa > sample-miniasm.fasta

minimap2 sample-miniasm.fasta sample.fastq > sample-map1.paf

racon -f sample.fastq sample-map1.paf sample-miniasm.fasta > sample-racon1.fasta

minimap2 sample-racon1.fasta sample.fastq > sample-map2.paf

racon -f sample.fastq sample-map2.paf sample-racon1.fasta > sample-racon2.fasta

#for additional explanation see: https://yiweiniu.github.io/blog/2018/03/Genome-assembly-pipeline-miniasm-Racon/

Supplementary data 2: New genome sequences for cauliflower mosaic virus (CaMV instead of KY810770), cabbage cytorhabdovirus 1 (CCyV1 instead of KY810772), tomato chlorosis virus (ToCV-1, ToCV-2 instead of KY810786, KY810787) and tomato yellow leaf curl virus (TYLCV instead of KY810789) used for the purpose of the analysis described in chapters: Analysis of Viral Sequence Content in Complete Datasets; Estimation of Accuracy of Obtained Sequencing Reads or Contigs and Viral Consensus Genome Sequences and Comparison of the Performance of

Nanopore and Illumina Sequencing for Detection of Viruses on Rarefied Datasets.

>CaMV

GGTATCAGAGCCATGAATAGGTCTAAGACCATAACTCAAGAGGGTAAAACCTCATCAAAATACCAAAGAGTTCTTAACTCTAAAGATAAAAGATCTTTCAAGATCAAAACTAGTTCCCTCACACCGGTGACCGACAGGTTTACCACCGTAAGGTTTCAGAACAACATCGAATGCGTTTACGCCAACTTCGACTCTCAGCTCAAGTCGTCGTACGATGGTAGATCTAAAAAGATCAAGAATCTAAGCCTTAAAAATCTTAGATGTTATGAAGCCTTCCTCAGGAAGTACCTTCTGGAACAATAAATCTCTCTGAGAATAGTACTCTATTGAGTATCCACAGATAATATAATCTTCTGTGTTGAGATGGATTTGTATCCAGAAGAAAATACCCAAAGCGAGCAATCGCAGAATTCTGAAAATAATATGCAAATATTTAAATCAGAAAATTCGGATGGATTCTCCTCCGATCTAATGATCTCAAACGATCAATTAAAAAATATCTCTAAAACCCAATTAACCTTGGAAAAAGAAAAGATATTTAAAATGCCTAACGTTTTATCTCAAGTTATGAAAAAAGCGTTTAGCAGGAAAAACGAGATTCTTTACTGCGTCTCGACAAAAGAATTATCGGTGGACATTCACGATGCCACAGGTAAAGTATATCTTCCCTTAATCACTAAGGAGGAGATAAATAAAAGACTTTCCAGCTTAAAACCTGAAGTCAGAAAGACCATGTCCATTGTTCATCTTGGAGCGGTCAAAATATTGCTTAAAGCTCAATTTCGAAATGGGATTGATACCCCAATCAAAATTGCTTTAATCGATGATAGAATCAATTCTAGAAGAGATTGTCTTCTTGGTGCAGCCAAAGGTAATCTAGCATACGGTAAGTTTATGTTTACTGTATACCCTAAGTTTGGAATAAGCCTTAATACCCAAAGACTTAACCAAACCCTAAGCCTTATTCATGATTTTGAGAATAAAAATCTTATGAATAAAGGTGATAAAGTTATGACCATAACCTATATCGTAGGATATGCATTAACTAATAGTCATCATAGCATAGATTATCAATCGAATGCTACAATTGAACTAGACAACGTATTTCAAGAAATTGGAAATGTTCAGCAATCTGAGTTCTGTACAATACAGAATGATGAGTGCAATTGGGCCATTGATATAGCCCAAAACAAAGCCTTATTAGGAGCTAAAGCCAAAACCCAAATTGGTAATAGTCTTCAAATAGGAAACAGTGCATCATCCTCTAATACTGAAAATGAATTAGCTAGGGTGAGCCAAAACATAGATCTTTTAAAGAATAAATTAAAAGAAATCTGTGGAGAATGAGCATTACGGGTCAACCGCATGTTTATAAAAAAGATACTATTATTAGACTAAAACCATTGTCTCTTAATAGTAATAATAGAAGTTATGTTTTTAGTTCCTCCAAAGGGAATATTCAAAATATAATTAATCATCTTAACAACCTCAATGAGATTGTAGGAAGAAGCTTACTCGGAATATGGAAGATCAACTCATACTTCGGACTAAGCAAAGACCCTTCGGAGTCCAAATCAAAAAACCCGTCAGTTTTTAATACTGCAAAAACCATTTTTAAGAGTGGGGGGGTTGATTATTCGAGCCAATTAAAGGAAATAAAATCCCTTTTAGAAGCTCAAAATACTAGAATTAAAAATCTAGAAAAAGCGATTCAATCCTTAGAGAATAAGATTGAACCAGAGCCCTTAACTAAAGAAGAAGTTAAAGAGCTAAAAGAATCGATTAACTCGATCAAAGAAGGATTAAAGAATATTATTGGCTAAAATGGCTAATCTTAATCAAATCCAAAAAGAAGTCTCTGAAATCCTCAGTGACCAAAAGTCCATGAAAATGGATATAAAAGCTATCTTAGATATATTAGGATCTCAAAACCCTAATAAAGAAAGCTTAGAAGCCGTTGCAGCGAAAATCGTTAATGACTTAACCAAGCTCATCAATGATTGTCCTTGTAACAAAGAAATATTAGAAGCCCTAGGTAACCAACCTAAAGAGCAACTAATAGAACAACCTAAAGAGAAAGGCAAAGGCCTTAACTTAGGAAAATACTCTTACCCCAATTACGGAGTAGGAAATGAAGAATTAGGATCCTCTGGAAACCCTAAAGCTTTAACCTGGCCCTTCAAAGCTCCAGCAGGATGGCCGAATCAATTTTAGACAGAACCATTAATAGGTTTTGGTATAATCTGGGAGAAGATTGTCTCTCAGAAAGTCAATTTGACCTTATGATAAGATTAATGGAAGAGTCCCTTGACGGGGACCAAATTATTGATCTAACCTCTCTACCTAGTGATAATTTGCAGGTCGAACAGGTTATGACAACTACCGAAGACTCGATCTCGGAAGAATCAGAATTCCTTCTAGCAATAGGAGAAACATCTGAAGACGAAAGTGATTCAGGAGAAGAACCTGAATTCGAACAAGTTCGAATGGATCGAACAGGAGGAACGGAGATTCCCAAAGAAGAGGATGGTGAACCATCCAGATACAATGAGAGAAAGAGAAAGACCACGGAGGACCGGTACTTTCCAACTCAACCAAAGACCATTCCAGGCCAAAAGCAAACGACCATGGGAATGCTCAACATTGACTGCCAAGCTAATCGAAGAACTCTAATCGACGATTGGGCAGCAGAAATCGGATTGATAGTCAAGACCAATAGAGAAGACTATCTCGATCCAGAAACAATTCTACTCTTGATGGAACACAAAACATCAGGAATAGCCAAGGAGTTAATCCGAAATACAAGATGGAACCGCACTACCGGCGACATCATAGAACAGGTGATCGATGCGATGTACACCATGTTCCTAGGATTAAATTACTCCGACAACAAGGTTGCTGAAAAGATCGAAGAGCAAGAGAAGGCCAAGATCAGAATGACCAAGCTTCAGCTCTGCGACATCTGCTACCTTGAAGATTTTACATGTGACTATGAAAAGAACATGTACAAGACGGAACTGGCGGATTTCCCAGGATATATCAACCAGTACCTGTCAAAAATCCCCATCATTGGAGAAAAAGCGCTAACACGCTTTAGGCACGAAGCCAACGGAACCAGCATTTACAGCTTAGGTTTCGCGGCAAAGATAGTAAAAGAAGAACTATCTAAAATCTGCGACTTATCCAAGAAGCAGAAAAAGTTGAAGAAATTCAACAAAAAGTGTTGTAGCATCGGCGAAGCTTCAACAGAGTATGGATGCAAGAAAACATCCAAGAAGAAGTATCACAAGAGATACAAAAAGAAATATAAGGCTTATAAACCTTATAAGAAGAGGAAGAAATTCCGATCCGGAAAGTACTTCAAGCCCAAGGAGAAAAAGGGCTCAAAGCAAAAGTATTGCCCTAAAGGCAAGAAAGACTGCAGATGTTGGATCTGCAATATTGAAGGCCATTACGCCAACGAATGTCCTAATCGACAAAGCTCGGAGAAGGTTCATATCCTTCAACAAGCAGAGAAATTGGGTCTCCAGCCCATCGAAGAACCCTATGAAGGAGTTCAAGAAGTATTCATTCTAGAATACAAAGAAGAGGAAGAAGAAACCTCTACCGAAGAAGATGATGGATCATCTACTTCAGAAGACTCAGATTCAGAATCAGACTGAGCAGGTGATGAATATCACCAATCCCAATTCGATCTACATCAAGGGAAGACTCTACTTCAAAGGATACAAGAAGATAGAGCTTCACTGCTTTGTAGACACGGGAGCAAGTTTATGCATAGCATCCAAATTCGTCATACCAGAAGAACATTGGATCAATGCAGAAAGACCAATCATGGTCAAAATTGCAGATGGAAGTTCGATCACCATCAACAAAGTCTGCAGAGACATTGACCTAATCATAGCCGGAGAAATATTCCATATTCCTACCGTCTATCAGCAGGAAAGTGGAATCGATTTCATCATCGGCAACAACTTCTGTCAGTTGTATGAACCTTTCATACAATTTACAGATAGAGTTATCTTCACAAAAGACAGGACATACCCTGTCCATATTGCGAAGCTAACAAGAGCAGTGCGAGTAGGCACCGAAGGATTCTTAGAATCCATGAAGAAACGTTCAAAGACTCATCAACCAGAGCCTGTGAACATTTCAACAAATAAAATTGCTATTCTTTCAGAGGGGAGGAGGTTATCAGAAGAAAAACTCTTCATCACTCAACAAAGAATGCAAAAGATCGAAGAACTACTCGAGAAAGTATGTTCAGAAAATCCATTAGATCCTAGCAAGACTAAGCAATGGATGAAAGCTTCAATCAAGCTTAGCGACCCAAGCAAAGCTATCAAGGTTAAACCCATGAAGTATAGCCCAATGGATCGTGAAGAATTTGACAAGCAAATCAAAGAGTTACTGGACTTAAAAGTCATTAAGCCCAGTAAAAGCCCTCACATGGCACCAGCCTTCTTGGTCAACAATGAAGCCGAGAAGCGAAGAGGAAAGAAACGCATGGTAGTCAACTACAAAGCTATGAACAAAGCTACTGTAGGAGACGCATACAATCTTCCCAACAAAGACGAGTTACTTACACTCATTCGAGGAAAGAAGATCTTCTCTTCCTTCGACTGTAAGTCAGGATTCTGGCAGGTTCTGCTAGATCAAGAATCAAGACCTCTAACGGCGTTTACATGCCCACAAGGTCACTATGAATGGAATGTGGTTCCCTTCGGTCTAAAGCAGGCACCATCAATATTCCAAAGACACATGGACGAAGCTTTCCGTGTGTTCAGAAAGTTCTGTTGCGTTTATGTCGACGACATTCTCGTATTCAGTAACAACGAAGAAGATCATCTACTTCACGTGGCAATGATCTTGCAAAAGTGCAATCAACATGGAATCATCCTTTCCAAGAAGAAAGCACAACTCTTCAAGAAGAAGATAAATTTCCTTGGTCTAGAAATTGACGAAGGAACACACAAGCCTCAAGGACATATCTTGGAACACATCAACAAATTCCCAGATACCCTTGAAGACAAGAAGCAACTTCAGAGATTCTTGGGCATACTCACATATGCCTCAGATTATATTCCGAAGCTAGCTCAAATCAGAAAGCCTCTGCAAGCCAAGCTTAAAGAAAACGTTCCATGGAGATGGACAAAGGAGGACACCCTCTACATGCAAAAGGTGAAGAAGAATCTGCAAGGATTCCCTCCGCTACATCATCCCTTACCAGAAGAGAAGCTCATCATCGAGACTGACGCTTCAGATGATTACTGGGGAGGTATGTTAAAAGCTATCAAAATTAACGAAGGTACTAATACTGAGTTAATTTGCAGATACGCATCTGGAAGCTTTAAAGCTGCAGAAAAGAATTACCACAGCAATGACAAAGAGACATTGGCGGTAATAAATACTATAAAGAAATTCAGTATTTATCTAACTCCTGTTCATTTTCTGATTAGAACAGATAATACTCATTTCAAGAGTTTCGTTAATCTTAATTACAAAGGAGATTCGAAACTTGGAAGAAACATCAGATGGCAAGCATGGCTCAGCCACTATTCATTTGATGTTGAACATATTAAAGGAACCGACAACCACTTTGCGGACTTCCTTTCAAGAGAATTCAATAGGGTTAATTCCTAATTGAAATCCGAAGATAAGATTCCCACACACTTGTGGCTGATATCAAAAGGCTACTGCCTATATAAACACATCTCTGGATCTCTGAGAAAATCAGACCTCCAAGCATGGAGAACTTAGAAAAACTCCTCATGCAAGAGAAAATACTAATGCTAGAGCTCGATCTAGTAAGAGCAAAAATATGCTTAGCAAGAGCTAACGGCTCTTCGCAACAAGGAGAAGACTCTCTCCGCCGTGAAACACCGGCACAAGAAGTAGCAGTTCACTCTGCACTGGCCACTTTTACGCCAACTCAAGTAAAGGCTATTCCAGAGCAAACGGCTCCTGGTAAAGAATCAACAAATCCGTTGATGGTTAGTATCTTACCAAAAGATATGAATTCGGTACAGACCGATAAAAGGCTTGTCACGCCAGCGGACTTCTTACGTCCACATCAGGGAATTCCAATCCCATATAAACCTGGACCTAGCAGTTCAGTTGCTCCTCTCAGAGCAGAATCGGGGATTCAAACCCCTCGTATCAACTACTACGTTGTATATAACGGACCTCATGCCGGAATATATGACGACTGGGGAAGTGCAAAGGCGGCGACGAATGGAGTCCCCGGAGTAGCACACAAGAAATTTGCCAATATCACAGAGGCGAGAGAAGCAGCTGACAAGTACACTACAAGTCAGCAAACGGATAGGCTCAACTTCATCCCCAAAGGTGAAGCCCGGCTAAAGCCCATAAGCTTTGCAAAGGCCCTCCTAAGCCCAGCAAAGCACAAGACGCAGTGGCTCACTCTTGGAACTAAAAAGCCCAGCAGTGATCCAAGCCCAAAAGAAGTCTCCTTTGAACCGGAGATCTCAATGAGCGACTTCCTCTATCTCTACGATCTAGGAAGAATGTTCGACGGAGACGGCGACAACACCGTCTTTACAACTGATAACGAGAGCATTAGTCTCTTCAATTTCAGGAAGAATGCTGATCCACAGATGATCAGAGAGACGTTCCAGGCGGGACTAGTCAGAACGATCTACCCCAGTGCAAATCTGCAGGAGATCAAATATCTTCCCAAGAAGATCAAAGATGCAGTCAAGAAATTCAGAACTAACTGCATCAAAAATACAGAGAAGGACATCTTTCTCAAGATCAGAAGTACTATTCCAGTATGGACGATTGAAGGTTTACTTCACAAACCAAGGCATGTCATTGAGATCGGAGTCTCAAAGAAAGTAGTGCCAAAAGAATCAAAGGCTATGCAGTCCAAGATTCAAATGGAGGATCTAACGGAGTTAGCCACCAAGACTGGAGAACAGTTCATACAGAGCTTGCTTAAGCTCAACGAGAAGAAGAAGATCTTCGTCAACATGGTAGAACACGACGTGCTGGTCTACTCAAAAAATCTAAAGGAAACAACTGCCGAAGATCAAAGGGCAATTGAGACCTTTCAAAAAAGGGTAATTTCGGGAAACCTCCTCGGATTCCATTGCCCAGCTATCTGTCACTTTATTGTGAAGATAGTGGAAAAGGAAGGTGGCACCTACCAATGCCATCATTGCGATAAAGGAAAGGCTATCGTTGAAGATAAGCCTGCCGACAGTGGTCCAGCAGACAGCTCGGGTCCACCCACCACGAAAGAAAACGTGGAAAAAGAAGATGCCGTTTCCACTACGACATCTTCAAAGCAAGTGGATTGATGTGATATCTCCACTGACGTAAGGGATGACGTACAATCCCACTATCCTTCGCAAGACCCTTCCTCTATATAAGGAAGTTCATTTCATTTGGAGAGGACACGCTGAAATCACCAGTCTCTCTCTACAACTCTCTCTCTCTACATTTCTCCATAAATAATGTGTGAGTAGTCTCCCGATAAGGGAGATTAGGGTTCTTATAGGGTTTCGCTCATGTGTTGAGCATATAAGAAACCCTTAGTATGTATTTGTATTTGTAAAATACTTCTATCAATAAAATTTCTAATTCCTAAAACCAAAATCCAGTACTAAAATCCAGATCTCCCAAAGTCCCTATAGATCTTTGTGGTGAATATAAACCAGACACGAGACGACTAAACCTGGAGCCCAGACGCCGTTTGAAGCTAGAAGTACCGCTGAGGCAGGAGGCCGTTAGGGAAAAGATGCTAAGGCAGGGTTGGTTACGTTGACTCCCCCGTAGGTTTGGTTTAAATATCATGAAGTGGACGGAAGGAAGGAGGAAGACAAGGAAGGATAAGGTTGCAGGCCCTGTGCAAGGTAAGAAGATGGAAATTTGATAGAGGTACGTTACTATACCTATACTATACGCTAAGGGATACTTATATTTATACCCTATACCCCCTAATAACCCCTTATCAATCAAAGAAATTATCCGCATAAGCCCCCGCTTAAAAAATT

>CCyV1

GATGCTTTTGTATCTTAATCCTTAAATGTTCCAATGTAGAGAAACGGTACATTAAAGAAAACAGGTGAGAAAGTCAACATAGACTTAAAACCTTGTTTCCTTTGTATCAGCGGTACCTCAGCCAATCATGACCAGTCAAAAGACAGAGCAGCAATTACAGGATGAAATTCAAAGAATAAGAATGGAAAGAGCCAGGAAAGGCAAGAATATTGAAGCAGGTCCCTCTAATGTGCCTCCGCCAGTCAAACCCAGAACTGTGTTACCAAAACAAACATCCAATCAAAGGTATCTGGAGATTGACTCTGTCAGTGTGGGGAAATTAACATCAGTTCCGTGGAGCGACACAGAGCTGTCGAAAATACCCATCTACAGAGTCAACGCAATTAATGCTGCGAAATGTCTCACACTAGGGAGGACTGTTTTTGAAAACTTGAATGCCGGAACTGTGACTGCAGCTTTGGCTGATATGTGTTTGGCCCTTGCTGTGTCTCTTCCCAAACCTGCACTTGCTACTTTCGAACACCTGTTGACTCCTATCCCGGCTACTATAGGGACGGGTGTTGCTTTCAATCAGCCGGAAGTGAATGATGCAGCTCCATCCCTTACCGCCACACAACAAATGGCACTGAACAGGGCGCGAGAAAGATTGCAGACAGAAACTGATGCAGAGAGACAAGCAGATTTACAGAGGACCATCGATCGGCTGGAACAACAGGTTAACGGCGAGAGAGTCAATGCTCCGGCGAACCATGTGAATGAAAGCGATGCAACAGCTTATTGCTTCTTAGCTGCATTTATCATGAAGCTAAACGGTAAAGCCGAGGACGCATTCCAAGAAGGGATCGCTAAGATGAAGATTCGTTATCCTGCCTGGTACGAAGGGGGTTCTCAGGTTCTACTCAACTTTAATCCGACTCTTGAGACTCTGAAGGCTCTGAGAACTATATTCAACAGGAGACCAGAGATCTTATCGACTTGGGTGATGACAGTTGCTGTCAATGAGAACCGAGAAGGTGTCATGTTGCCTACCCATCAAGGTCTGCTTAATTACCTGGTGTGCCAGCAATATTCTTATTTCGGGATGCATGCTTACTCGCTGTTGCTCAGTATCCATGAGGCGACTGGAATAAAACTAGGTCAGCTATTGAGAGAGATGGATTGCCCTATAACTAGAGCCGGAGTGATGGCTGCTTTCGACTTGATTAAGAACCATGAGATAACATCTAAGAACCCCGCTAGAACTACCTATTTTAGGTATGCCAGAGTGTGGAATTCAAACTACTTCAGAGCTCTGCAATCCTCTAACTGTACAACATTGGTGTATGTGGCAGCTAAGGTGGCAAAGATAACCTCAGCACAGAAAGTCGGAGGAGATCCAATGGAAATCTATGCTTTAAAGAACATAGATGAGGTCATGCTAACAAGGCTGAACAAGGTTGCTGCTAAGATGTCTGAGTTAATATTGACTGCTATGATGGAAGATGAGATTGCTGGCGTTGCCTGGCAATGACCACTCAATGACATTATTATTCTGAATGGTCAATCTATGAGGCTATCATAGGGTTTCAATAATTTGTGTGCTGTGAGATAATGCGTGCTCTCTATAAACGGGTTGTTTGTTTAATACTTATTTAAATATGTCAGTTTGTTATGTATCCATAGTTTGAGATTTAAGAAAACGAGTGAGACCCACAGTAAATGTTAGTCCTTTTGTCTAAAGTTCCTGAATTCTTGTGGATCTATTTCGGTTTAATACAATGGATCCTCAACAATCTGCTATCGACTTCTCCGAGATTGATGCAAATCTTCATCGTTCTCCTTATGTTGTCCCCGCTTTGGATGATGATATGGATGGTGGTGAGATTGTACAGGATGATCTCAGTGTTCCTCGGTCTACTGATGATGTTGCTCCGCCTACTCAAAAATCTTCTCTTGTGTCCTCTGAGGTTGTAGAGTCACTTCTGTATGAGGCATCTGTCCTCCACGGTATTGCGGTCACACCGCACATGTCTACTACAGCCATTGCATTGGCACACAACATTGGGTTGGAGCCTCACTCTTTGGATTGGTTCCTGGCCGGTATATCTTATGCTAACAACAGTATGATAGTTGAGAAGCTGTCATCCATCATAAAAGATATGCAAATCGAGACTAGGAATCTTCAGACTGCGACATCAAGTGTGGCTGCAGTTTCAAATGAGTTTTTAGGGAAGATGAGCCGGAACAAGAGAGAGATCATAGACGAAATGGAGAAAACAAGAGAATCCGTGTTGAATGCTGTAGCTTCCATTCAGTCAGCAGAATTGGGATCCCACATCGGCGATGACTTTGTGAACTTGACAGTCAATCCGGAGGGAACATCGGAGAGATCTACAATCCCGCCTCCGGCACCCGTTGATGCAGGACTGTTGTCGAAAGTCCTATCAAATCCGGTGACTTTGAAGAGCCCGGATGAGATTCTATATGCTCGGAAATACAAGCTATTGCTGGATCTAGGCATCGAGATACCAAAGGCAAACTTATCTCCAGATATCATGAACACTCTCATCCCGGATTGGCAACTCGAGGCGGCCGAAGCAGGGTTAGACTCTAAGACCAAAATGGAGCTGACAGATGAGCTGCTAGACATTGTCATCACCCTGAATCTCATCTGAGGTGGTGTGCTCATCATGAGCGTATGCAAACCCTTATGTATCATATGTGTGCCCTATAAAGAGCGTTTGGTGTTGCTTTAATATCTTAATGTCTGCTACAATTAGACTCACAAAGAGTCCTACCTATCGTCTGCTTTGTATTATCAATATTATCTATTATCTTGTTATCAATATGTTAGTTCTTAATTGTCAAAGTATTTAATAAAGGTAAAAGGTTCATGGTTTGTCTTTGAAAGGGTTCCCTGTGTCTTAGTTGTTTGAAGTATGTGTGGAATTTATGCTTAACAATGTGTGATTTAAGAAAACTAGTGAGAGTCACAATGATCAATGTTAATGATGTACGTAAGCAGATAATAAAATCAGGATCATTGACTTCAGCTGTTGGAACTGGATACGTCTATGACGGATCATACAATAAATATGCTAGGAAGAGAGAACTCAATCTTAGAGTCTCATCATCAGGGGACAAAAATATAATGATGAGACATGTCCCTATTTTTGATGACGAGGATCTCCGAGCACTCAGATCAGAAGCTAATTCAAACAAATATATACATATTGGGTGTCTCACCATATCTATTGAACCCTTAATGCATAAGAGGTATATGGACTTATATGGAGATAAAATGAGGGGGATATGTGCTGTCATAGATACAACATTCGCTGATCCGGCAGAGTCCATAATTTCAGCTCATCGTTATGAGCTATCTAAAGGGAGAGCAGATTTTGTGAGCATGCCGAATCATTGTCTGTCTCTGTTAGATCCCAACTTAAAGCAGAGGTTGTCAGTCATGATTAGTTTGGATGGGATAAATGTCAAAAAAGGAAATGAGATGTTTAACGTATGTATAGGGTATATAGTCACAGGTGTGAATACCCTAAACCCTACTAAGGCAGTCTCTATGTCCAATATTCCTATCACTGGTACGTCCGAATGTGAGCCTGGCGATTTAAGTGAGGATATGTTAGAAGGAATAAAAGGTTCAAATGGGAATCTTCTGATATCCCACGACCCTTCTGATGATGATATATACATAAAATCAAAAGGATCTTTCTTATCCAACTTGTCTGGGAAAACAAAGATTATTAAAAGGCGAACGATGAGAGCCAAACCGATAGACATCACTCCTGCTGATCATAACTCTAACGTCAGAAGTATTTCTAGTGATGAGCATTTAGGAGAGTCAGAGCGGCGTTCTTGTTCAAAGATATCCGCATCAGATTGTTATCAAACCTTGAATCATCAAGCTGTATTGAAGAGAGCTATGAGTTCCAGATACGGGTGATGCTAAAGCTTACAGGGTCCTGACGCCTCTTAATAAGAAGGAAAGGATCATTCCTTGAATAAGATTTAAGAAAACTATGAGAATCTCAAAATGGCTTTATCTACTTTCCATTGGTACCGACTGACCTTTTATGATTCGGTCATCAGCTTTGACATCCCTCATGAGAAGACCATCGAGGATCAGAATGCTGCCACTTGTCCTGTGTTATACCAAACAGCTATCTCAACTGCCTTGAATCAGAAGTCAGGACTCGTGGAGATCATGAAAAACTTAGAGAATAAGAGAGTTATCACTCATGTCTCTATGGTTGCTCACTCTGAGTTCTTTGGACCCGGGACGATAAGATGCTCTTATGTTTTCCCAAATGAAGTGTTCATTCCTACTCGCCACACACTGGATCATGGCCTTGTCAACTACACAAAGGAGAATGCCCTCGTTGCGTACAAGGGTTTAGATTTCATAGCAAATGTGATGTTGAAGATAGGCACTGCAGAGGTGTCACCAGGGGACATTAAAATGCTTAGGGGCGCAAAGCCGAACAGTTTTGTCGGATTCATGGATGATGGAAAGGCAATAGTTCCCAAAAAGCCAATATGATCCACTCCCTAGAGGAAACTCCCTTTAAGAGATGGGTGCCCGATTTAAGATCTGATTTAAGAAAACTATCGAGACCTACAATAAACATGGGTTATTTTGCTATCTTTGCGTTGTGCTTTGCCTCTCTCGTTTCCTCTGGAGTACATGCTGGTTTTAATCATTCAGTAGGACCAATTGCCGTTTGTAAAAAAGATATGCTAGACAGTCGAGCATATGTTGAGTCATGTTTCCAACGATGTCAACGGCCAGAAAGACCATCCGGACACGGGATTCTTGAAATATTCTCGGCCCCTCAGAATAAAAAGGGACCGTCTGTGATTCATTGCACCAAGGTCAGACTATCTCAGACTTTTACAGAGACATGGTCATTTTCTCAGTTCAGCGGCCCTCTCGAGAAAGCTCTTCTTCCTTTGAGTGTCAAAGAATGCCAAGATGCTATAAGAGAGAAATGCCCAAACTGGAATTGCAATGTTCGGTCTCCTGGACAACTTGAGGAAGAATATCATTATGCCTCAGAGACCACAGTCACAAAAGATTACTTAAATCTTGTGTCCGTTCCTAGTGGGTTGACGTTTTATGACACTGAAATCAAAGTGATTCCTATGGAATCCTCAGCGTCATTCAATCTCTCTGCAGAAGTGGGCACAGAGAAAGATTCAGTGTACTTATGGAAATCATCTGAGGTGACAAGTTGTCCTTATGAGAGTGCTGTATCAGTTGGTTGTGATGTTTACAATGGAACTGTAGATTCTTATGTCTGCAGGGGTAGCAGGATTTCTGTTGAGGGGATATCTCGTGTTAAACAATTAAAAGGGTTATGCTCTGATGTGTCCCGAGCTCCATCAGGATTGCTGATCAAGTTCACGGCGAAGGAGTTAACATCTGACCGGGCAGACAGGAAGGTGTTCTTGACTCAATCTGCATCTGAAACTACCATCGAGACGTCATTGAGGACCCAAACAGGAGATGCCTTGTCCGTTATAGATGAGGATCTTTGTCAACTCCAGTGTGAAATTCTTGAGATGAACTCCAGGTTAAACTTAGGAAGAGAGTCTCTCATGAGATTAGGAAACCGTTATGTCTTGCAGTCCAAGACTGGTTTTATAAGGGAATGTGATCCGCTGGTATCTTGTGCGGTCACTACTCCTCACCTTTATTGTGGCAGCCCTGTTAGAGTGTCTGTGTCATGTGACGAGAGACATTGGATGTGGAACCCCATGAAAAGTTATGTAGAGGAATCCGGCGTATGCCATGCTGTGGAGCGGCATGAGAAATTACATATGCTTTTAGGCTCTCATATCTATGATGTTGGAGATGACCTGTCCATACAAATCAACCAATCTGATAACGTAGGGCTACCACATGACCTCTTGAATATCAGAGCTTCTACAGTCAAGATGGAGACATTCGATCCTGAGGCATTGAAAAGATCATGGGCTTATGAGTTGAATAACCCAAGAGTTACATCTCAATATGGTAATATATCTAGAAACATAAACCACTGGGACGTAATGAATGAGATGACCACAGGATTCGGGAGGGTTATGAGGACAATCGGTGATGTTTTCCATAAGACTTATCTATTCGCAGGGATAATAATAACTCTGGTCTTGGTCTTCTGGGGATATGATACCATAGTGAAGAGGGGATGGGTGAGAAATTACAGAAGGGTTGCTAGAAGTCCCACTAGGCAGGAACCTACTGGAGAAGCAATATGGATGTAAGAGAGGTAATAAAGGAGAACGCAGGGGGATTGATAGTATTAATTCTCCTTAAGATCTTATTGTTGTTCTTATGCTGTACCGTACGTTCTCTGAGATGGAGACGAATAAATAAGAGATTCCACACAGCTTTGTGGCATGTATGATTTAAGAAAACTAATCTGATTAAACCAGTTGAGACTTGCAATTGACTTCCTTTTGATCAGATACTATGAATTTCGAAATAGAATCAGAAGGAATCAAAAAGAAACTTTATGACCCGTTGCCCGACTTTCATTTGCAAAATCCGTTGTACTCACTGAATGAAAGGATACAAGCCTGGAAGAATAAGAAGCGCTTACCTGTAAGATTGTATAAGTCATTCCATGCTCTATCCTCTGCCTCCAACAATCTGGAAGAAGGAAACCCCATAGATCTCCTCAATCTCTTTTTATCACTTCCTGTCATATCTGTACACCCGTTACCAGCTTATGCTCTTGATGACTGTCTTTACAAGCTGAGATTAGATTCCAAATCAGACCATAACATCTCTACGAAGCTGCTGAAGGACTGCTGGCTGAAAGTATGTGCCAATTTCCCCGCAAGATTGTGGAGCTCTATGAGGGAGGGCCAATTGTTGCTATCCGGACTCAATGCTCTCTCTAGTCGAAGACCGTTACCATCTGGGTTTGTCAAGATCAGCGAAACATTATATGAAAGGAGAACACATTCTGTCAGATGGTTGATCAGCCCGGCTTTATTGGGTGTCTGCGTGAACAGATCAAACAAGATCAAGCTGATTGACTCAGATTGGTTGCGATCGGTAGTAGATGTGTGCACAGAGAGGTTCTTAGTGTGTCTAGGCCTGACTCTCGGATCATCATACAATCCTGATCATTACCCTACTTGGGATCGCCTATTGCCAGTGCTGACATGGGGAGATGACGTGCTTAAGAAACATGGTAATTCTGGATTCAAACTACTTAAGGCATTTGAGGCTATAGTACTGGGAGTCCTTCAGACCAAAAGCACAGGGACTTTCGTAGACAATCACCGATTTCTGAATAACACTCTTGCAGACCTAATTGATGAAAATGTAGATTTTGGAGTCAGAGCCAAGAGCTTGGTCTCTTACATCAGTCAGTTTGAGTCACCTCATCATCTCACTCAGCTCTATGGCCTTCACAGGATCTGGGGACACCCCATAGTGGATCCCGCTAAAGGAATGATAAAGATGATTACAATAGGCCAAAAGGATATCATAGAGCCGGGACCTCTCCCTGAAGTGTTGGGCGTTCATTTTAAAAAGATGTTCATTAAGAGTTACAAAGACAAGAATGGGGTTTATCCCAAAGTTAGAGACCAAGGAACGACTCTGGAACAATTGTTATTGGGAAATCAAGACTGGGGATTGTGTTCTAGTTTAGACTTGGAAGGTCAATGGTCAACTTTAAAGTTTGAAAAGAACTTTGAAATACCCGAATCTTTCAACCTGTCTATGATCGTGGCCGATAAGAGTGTGTCACCAACCTTGAATGAGCTGAAAGCAAATGTATTGACCAGGGGAACGGTGATGAATAGTGAGCTGAGGAGGGGGGTCTTGAGGTGGATTAACAGCGAGTCAATAGACCCAAGAGAATTCTTGAAAGATACAGCAGAGGGCAAGTTTCCTCATGATCACAAGATCATCGGTTTAAGATCCAAAGAGCGTGAACTGAATCCAACACCTAGGATGTTTGCATTGATGTCCCATCTCATGAGGGTGTATGTAGTGATTACTGAATCGATGCTATCTGAACATATCCTCCCTCATTTCCCTCAGATCACTATGACGGATGATTTGCTGAGCTTGACAAAGAAGACATATACAACCGTGAGAAACCAGTCCGCAAACAAAGCCAAGTCGAGGCTGATGGCATCCAAAACCGTATGTATGTCGTTGGACTTCGAGAAGTGGAATGGCCATATGAGACAAGAAGCTACTCTTCATGTCTTCAAGTCTTTAGGAGAGCTGTTCGGCCTAGATGACCTCTATCATGTGACCTACGATATCTTCAAGGAGAGCTATTTTTATCTAGCAGACGGGTCATATGTCCCTGTGTTTAATGCAAATGGGGATTTTGCCCCCGAGCCACCATTATCGTTTACTGGTCACAAGGGTGGACAAGAAGGGCTGCGTCAGAAAGGTTGGACCATATTCACTGTTGTCGGCCTAGACAAAATCTGCTCAGAGCACAACTGTTCATATAAAATCATGGGGATGGGAGACAACCAAGTGTTACAGCTGACTATGTACACCAACAAAGTTGACATTCAAGGAGCCCCCACTGAACAGGGCATGAAAGATATGCAGAGGACTTTGAAGAATGTTTTCTCGGATTTGTTAGAGACCTTTAACTCGCTTGGTTTGCCGTTGAAGCCGCTGGAGACATGGATATCAGAAGATCTGTTTGTCTACGGGAAATACCCCGTGTGGAAAGGAGTTCCTCTGTCCATGGATATCAAGAAGATCATGCGGATATTTTCCAACTCTAACCAGGAAATGATGACTGCTGAGAATATGTTCAATACTGTTGGAGGAAATGCTCAAGCTGCGACCCAAGCAAGCCCTGTTCTAGGTGTCAGCTACATGATTGGTCTCTTTATGATGTCTGTGTGCGCGGATGACTTGCTGGATTATCACCCGTTGTTGGGAGAGGGGCTCTTGAAAAGCTTGGCAGATCAGCCTGAATGGGTAATAACGATCAAGAAAGAGAAGCCACGAAAGACTAAGCTGGGTACTTGGAGGCCATCTAGGATGCTGATCAGACGACTTATGAGTATGGTCCCTCGGGTGCTTGGTGGGTATGTCTCCTTCAACTTGTTTGGGTTGTTAATGAGAGGGTTCCCCGACCCTGTCTCTCTTGCGTTGAGCCAGCTTTATGCATACGGAGTTAAAGACGCTGATGATGATGCTCTATTAGTTATCCTAAAGAGGTGGTGTGATCCGATATACATGCCAGACGTCTCGCTTAAATTGCTCATAGAGGATGTGTCTAGCGTCAATCTTTTGGCGCCCGTAACACCAACAGCCGGGCTAAGAAGGGTCGTGGAAAAATATCTGGCAGAAGGAAGAGCTATCCGCAACCAAGAATTCAAGGATCTGATGAAAACCAGGGTCGCCGATGTAGAGGATGTCATTGCTGACCAGTTGTGCAAAGGTGACACATTACACATTCGTTTGTTGCATGACATTATGGAGGCTACGATATTTGGTTACATTAAGAGCATAGTGAGCAAGGTTACTAAATCCTCTACCATCTTGAGCATCGCAGTAGATAAGTCTACAAGAGATCCTTTGGCTAAAGTGATAGAGGATGAGCGGAACTACTTTAAATTCTTCGTTTGGCGATGCTCAGTGGAAGGGGGGAACCTTCTACCAGATTGCCCCACAGATTTGGCCAAATCAATGAGACTGAATGGTTGGGGAAAGTCTCTCATCGGAGTCACAGTGGCATTCCCTTTATCCTATCTTACCAAGACGACTTGTTATCGGAAAGATCAAGGCTGTAATTGCGAGGATGGTTTCATCTCACTCTATCTTCCTGACAACAATGTGACACCCAAGGAGTGGAATACAGCAATAGGGCGGAATCCCCCCTATCTGGGCAGCATGACAAAAGAAAAGTTGGTCATTTCATCTGGTGCGAAGATATACTCGGGAGAGCCGTTGATAAAACGGCCCATTGAACTAATGAGGGTAGTTGGTTGGTTTGTCCCGGAAAACAGCAACACAGCAGACATAATCAGATCATGTGTTGGAGCAGTGAGTGATCTCAATCCTAATGAGTTCAGGGGGATAACAGAAGGCAGCAGTGGTTCCGAGATACATCGATACAAAGACTCGAGTCTCAAACATGGCGCTTTGTGCTCATCTAATTACTTATATTCCACGAGATATCATGTATCTACAGACACGTTCTCCAGATATGCTAAAGGCTCCCAGAATTATGACATGATGTTTCAAGCGAACCTCTGCTACATTGTGGAGAGCACACACTTAGATGTCATCGATCTAAATGCCTCGGGAGAGATAACACCCAAGGTAACTCATTTCAACCAATCGTGTTACCATTGCATTTCTCCTTTAGATGAGACTTTTCATGATCTAAGAGATGGAGAAGCTGCTCGTGTGATCCCGTCTAACAAAACCAACAGATATTTGTATGTCAGAGCCGAGAAGGTTTCCCTCAAGCTGTATCTTCCTCCGTTCCCTGGATGGATAACCGGAACAATGCCACCGTCAGAGATGCTAAGGATGACGAACCTAGAAAGGAAGACATGGTTGGTTGAGAGTGTTGTTGACAACATCTGCATTGACATCCAAGGAGCAACATCAGAGTCCAACTACTTGACCACAGCCTTATTAGACATCAAAGAGCACAACAGATTGTTTTATCTTTCTGTGTCACCCGAAGCTGTTTATAACACATTGTGTTCTCGCATGATCATGATGGCTGAGTGGCGATGTCTAACTTCTGCTGACTGGAAGGTTCCAACTGCCTCGAGCATTGAAAGGGCTATCACAGCAATGATAGGGGATATGAACACGGACAAACTTTCTGGCATGGCGGGATTCTTCACGTGGCCCGAAGCTATGAAGCGATATTATTTTGCCAATGAAATTGTGGAACCGGATACAATCCCGGTCAATGTTGCATCTGCTTGTAAAGCAATCAAGATAAGCCTCATCAACTTGTTGTCATCAGGTAAGACATTTGACGTAAAGAGACAACATTATATCCTGCTGGAAGAGACGAAAACATCAAAGTTAGTTCTCAAGATGATGATTTATGAGATCCTGAGAGCCAGAACGTCCAGATGGTGTTGCTTAAGGGTGATTGGAAACATGTCCCCTTATGACTTGGCAAATTCTAACGCTTCGTTACTTACCTGTCACAACAGTCATGTCCTATTTCCGAAAGGAGTTGAAGGGATGATCAACAGAGCTCAGATTACGATGGATGCTCTCAAGAAGAGCATAGACAGCGAAGAGATTCAAGACACCTATCATGTCAGGAGAGAACTAATCCAACCCTTGTTAGAAACATCATGCAGGATAGGCTTTTCATCAAGCTTATTTAGAGCTCAACTGATTTTGAACCCCAATGTTGACGATCACAGATTTGTATCAGTGAGGCCCTCTGGAGCTGGAGATCTCTGCAAACTGTTCTCTTTACCTACAGGGGCTGAGTACAAGTACACGGATATCGTCTCATTCCTGATTCATGATATAAAGAGGTTGAAAGGGGCACTGGTTCTCGGGAACGGGCTAGGGGGTAGTAGTAACGTCCTGAGACGAATGTGGAGAGGGAAACTGATTATCTCTACCTTGGTAGATACGGGAGAATCAATTCCGCAAGCATACCCAATGTGCAACAATGCCTTCAAGTTCAGTTTAGATCCGGATGTCGACAGCAGCAGTATGATTAATAGAGTCAACGACATATCTCATGAAGGCTGGGTTAAAAGCTGGGCGAACATCATACCTCCGGATGTTGATTTTTGTGTTTCTGACATCGAGATAATCAATCCGACCCAAAACTACGACAGGAATCAAGTGATGAGGAAGGTGTTAGCACTTAAGTCTTGGAAGATGTTATTGTTGAAAGACTATGTCTATTCTGCATCAGAGTTAGAAAGCCGCTTGTCTATTGCTCTTCAGCATTCAGATGATGTTAAGATGTTATTCAGTGGAGCCAGGCAAAGAGTTGTTCCTGAGTTTTGGTGGGTGATAAAGAAAATGAAACAAGATCCTTTAAAAGCAGAGATAGGGTATCACAAAAGGGTCATGCAAGAGATATGGTCCGATTTCGAACATCATCTCAACTACTCAGATCCTCTCATTCCAGAAATCTTGACAAACATTAATCACATCCTCATGGACGAAGGGAGACTTGCAGCCATAACAGGACGGATAAAGCTCTGGGCCACTCTTCCTATAGCTGGGTCTGCATTGCCTCACAAGGGGTCATATACGAGGTTCTTTGGTTACTTACAAAGGGGAAAGAAGCCTGCTGATATACGATGGGAAAAAGATGACCTAGGAAGGAAGTTGTATATGAGTGATTATGATCAGCTCAGGGAGATTTTGCTAGGACTAGCAGCTTCCATGATTGCCCCTTTAGACAAGAGAAACCAGTTTGTCGATTCAACTCAGTACTGGGCTCTCATTTGGAAACCTTCTAGGACTGGGATATGGGTCCCTATGTTGAAGAAGATGGAGAGATCCTTGGCTCCGGCTCATATCTATGATATGGTCCCAGGATTATCTATAATGATGAGGAGAGACAGGCTGCTCTTTAAAGAATATGGGAACACAATAGAGTTCAAGCCAGATAGAAACAGGAAGAAGTTATGTTTCCCGATTACTAAAGCAGCCTTTATAAGGATTGAGAGGGAATTGTGATATTATCTGAAGCGAATGTTGTGATCCAAGCTTTATCCTAAGAGGGGGCTTTAGTTAATTATCATCAATATTAAATTGGATTTAAGAAAACTATATTATTTATTTTTAAAAAATCTTGGAATTATGAATTCACTGTCGGATACTCTGTAGGTGCATCGAACATCAGACATAAAACTCAAGGACAGTATGTTCAGGAGAACATTGGATCTTTGCTCTTTTGCAATTGTTAATGATTGAGTTAAACTCTTGTTTTCTTTTATTGTC

>ToCV-1

GAAAAATAGTATTCGTGTGATCACACAAAGTACTAATTAACTTAGCTTTAAGGCTTTCTGGTGTGTTGCCAGTTTGCCTGCCCGGTTGCCACTGTGTTCAGTGTCGACCTTATTATACCAGCATTCACCGCCATAGCATTACTAGAAACAGCACGACGAGTACACTAAACTAATCTGCCTTGCTTTACTTTGCGTTCTCCTTTTCGTCTCGGAGAGCGCGCCCCTTGCCTGCCGTTGTGGGTTGTTAGTGTTCACCATATAACCCCCTCTTGTTGTGATTTAGTAGTTTGTATTTTAGCTATTATGGATTCTCAGCAAAACCTAGTTTCGTTTAACGTAGATTTTAATGAAAAGAAAATAAAAGATACGTTTAGAGTAGTTAAGAGGCATATTAGTAATAAATATAATAAATCCTTTAAGAAGAGACTGTTTTTGTGCAGTTGCGATTTAAATGTCTCAATTGCCGCAAACTCAGTGTCGACTGCTCAGGGATGCAGTGTGAGGGCTCGGATAAAAACTCGCATGAACGTACTTAGACGGTTATGTGGGATACCGCATTGCAATTTCAACAAATTACCAGTTTCTGTATACAGAAAATTCGGTCACGATTTCCATAGAATAAATTCTGCAATAGACCGGTATTTGGAAAGTTCTGTGGGGTCAACAGGTGGAAAATCTCTCGAGGAAATCGAGAGAATAGATGGTTACACGGACCATGGCGATGGTTATCGTGTAAAACTTTACTCTGACGTCAACATTTGTGACGTGTTTGTAAAGTTCAATGCTGATGTTGCCACTGGTCACGATTTGAAGATCAGGCTCCAAAGACAGAAGAATAGAATTACCGGCCAAGTTAGGACGGTCGTTCATGCTGCGAAGAATGGTTTTGGGCTTGATTTTTCTCTGTGGTGTGATAGTTTCCTTCTCACAGACAGTAAAACTTCCGGCCAGAAGATGGTTATGGATATGATAGCGGCTGTTGCTATGAAGGTTCCGGATGTTTTGCCTGGATTTGGCCGTTTGTACTCCAGAGTTTTTAGTAAGAACTTTAAGCAGATCAGATCCGCTTTTATCAGAGAGTGCAAGAAGTACATGCAAACTTCTTGTGATAAGGCTAATGCAAGTCACCACGAGAAAGTGACACTCAAAGTGAAGCCGTCAGTCGCGGTGAAGAACGTGCCAGATGTGACCCATAAGGTCGGACCCGATGGTGCAGAATCGTTCGTGGTCACTTATTCTGATGGGAAACAACGCATAATTGTGAATGATGACGGTGCCGTTAGGAATTTGTTTAACGCTACTCTCACCAACGGTAAATATTTCATTCACCCGAAGGCTATGATCCCAGATAAAAGCTTTTTCTCGTCCAAAACAACTGAGTATTGTTGGTTGAATGCCTTCGCTGCCGTCAATAAGAAAATACCAGATTTCGTTGTACCTTACCCATGTCTTAGGATGCGGGTGTTGTACAACTGTGGTTTGGGCAGTGTAGTTGAGAAGCATTGTAAGTTTGTAAAAGCCGGTTTATACCATTTTGATCTTAGGTACTGTGCGCCTGAAAAACCCATCAATCTTAATGGTTATGTTGGTTCCAAGGTTGACACTGATATACCGTCCCTAGGTAAGAACATAAATGTCATCTTTGATGATTTGATCGGACATTATGTTCAAGGTACTAATTTGAGATCTGACAATCTGTTATCTAGCAACATCGTTAATAGATTGTCTGACAGAATAAACACAATGTTTAGCAAACCGAAAGATCTCTCGATAGCGACTTCGCTCACCGCATCAGAGAAACGTAAAGTGATCGATATGTTTCCTGAGTTGTGTTTGAATTTCACCGACACATCTTACTCCTCTCATCCTATTGCCACTGCAATTAGGTGTTGTGAGAACTTCATCATGGCCAAACGATGTGGTAATGAGGATTTTATTGATGCTGGTGGTGATGTTGTGCACTATATGTTAGAGAGTGTGAAAAACGTTCACGTTTGCACACCAATAGTTGATACCAAGGATGCCCATAGACACATTTCGAGGAGTGCTATGCTCGATACCATGTGGGGACTTAAGGACAAGGTGTCTTTCTGTGAGCATAAAACTGAAACTTGCACAGTGGAGAAAACTAATATTGTAGCGGTGGAGGTTTATGATATGACTCTGAAACAAATGGCACAAGCTCTTCTGTCACATAAGGCCAAAAGGTTTGACTTCAGCCTTATAATACCACCTGAGGTGTGCGACCCGGTGTGTGATGTGTACCTGCTTAACAATAGCCTACACGTTACCAATAACGGTGACAAGATTGAGTACGTATACGGCGACTTTGGTGAGAGTTATTTTCATGATAGAGAGAACTTGAGAGACATTCTACGAACTCAAATGTTCGTTTATAATGGAGTTGTTTTCAAGAAGTCATTAGAGTGTTCTCGTGACAATCTGCATTTCTTCTCTGTGGTTCCTTGCTTGGGCATTAAACCGGGTGTCTACACTTTGTCAACTCATTATAAAAAATCTGAGTCTGATAAGATTGAGATGACAATACCCGTTCGGGAGAAACTCGGACAAGTCGTCGACAAGCGGACGAGTGTTGATAGGGCACTTACTTACAGCTTGATAGAATATGTCATGAACACAGCGGCTAGGGTGGATGAGAAATCCTCTGACTATTTAGTTTCTCAGTACAGGGCCAAGAAGAGTATGACCATAAAAGGTAATAAAGTGGTGCCTAACGACTGCGATTTACCCGTGGAGTTGGTGCCAGGTTACCTAGCTATTATTTTGGCCGAGGGATTGAGGCTTAGGGAGAAGATTCAATACTTTGCAAAAATTAGTTATCATCGTCACTACAGCCCGTCGATTTTGAAGATATTCTCCCTCTTGATACAAGAGTTTGTAACGTTCGCTAAGGCTAAGTGTTACGATGGGTTTGTTTGGTTCCTTCGCAAGTGTCTGTCTGATAAGGTACTTGATAAGGTGATATTTGGTGAGAGGCGTATCCATGACTGCCAGTTCATAATGCGGTTCAAGCAAGAGGTCACTGTGGTCGGCGAGAGTGGTAAGCATTCGATCCTCGGTGATTCTATCCACGCCTTCACTGAAGCAAGTGAGAGAGCTGAAACTGGACTGGCCGATTTTGTGGACGGTAGACCGGATTTGTTCAAGCCAGATGACTATGAGAAGTTGTATGATCTAGTTCATTCTGGTGGTGGAAACGCAGGGTTTTTCAGTAAGACGGTTAATTATTACTCTCCGTTTTCTTTGTATTATTCTATTTACAATTTTTTCTCGAATTTTACTTCTTCAGCTTCTCGAATTTCCTTGTATACCAATTTCTTTATTTCAGTTATAGATTGGATGCGTTATCACTCTATCTCTTGTTTTGAATATTTTAAGGAGGTCGTAAAAGTCATAGTTTGTGCTGTTATGGGGAAGTTGAAATCAGGTATGCAGTCTCTTTGGGACAAACTCAAGCGGTGTGCTAAAGAGATGAAGGGGCTCGTGAACAGCGACAGGCTGTCTTTGGAGAAGAAACTTGATGATATCTTCTCTGCGGACGACCAGATTGCTGAAGCATTGTCCTCGGATGTACAATCTATTGTTACTGAAGCAGTGCTTTCAGAGCAATTGATCTCGTCTGGAGGCGGTGGAGTCATGTCACGACCGATGAAATGCGTTCGTGAAATGTACGATCATTCACGGCGATCCTTGCGGTGGTTTTGTACTTGGTTTCTCCAGCAATTTAAGCTTTACAAAAGAAATATTTTGTACATTCCTGAATTTTTAAGGGAGTCCTTTGATGAGATCTTTGAGGCCCTAAAAATGAAGATTTTGTCTAACGATTTCATCGAGATGACGATCCAAGGTATGTCTTTCTTTGTGGTTAATTTAACTACATCGTTGTTTTTTGGTAAGATAGGTTTAGGTTTGAGTGCTGTGTCTACTATTTTCTATCTTTCCATTAAATACACTGGTTTGGAGAGAAGATTTCTTGGTACTTCATTTGTCAATGAACATCTCTCTTCCGCTATAACGACTGGTGGTTTTTCCCATCCGTACATGATTCCAGTGAGGTCTTTGGTTATGAAAAGTTGCCAGAATTGGATGAAACAGAAACTGTTGAGGTATGAGACGGTCTCTCCAATAGTTACTGACCTTATTGCTAAGGACGTGTTAAACGCGTCAGTTTATCAGTATGTAACTCCCTACAGAGTTAGAATGGGGTTGTATTTTAGTATTTTGTTGGCCCTACTGAAACCGACATTTGCTGGCGTGCTCCTGGTGTGTATTCTGTTGGTGGCGGAACATGCAAAATTTTACAGAACTGTTGTTGTTCAAGCCAATGTTCATTTATCCTTTGCTTCTCGACTGAGGAGATTGAATCCTACTTCGAAGGCTAGAGCGATTAAGCAATTACTCGTTTCCAAGTTTGATAGGAAGAGATTTAGCAAGGATAACAGTGACGATAAGTTCTGCGATACATTTGAGGACTTTGATTGCGGTCCGACCGAGTTTGGAATAAACTCTAAAGATTTGGTTGGTGCTGGGGAGGCCTCGACGTCTGAACTGCCTGTGGTAGAGTTGGATTACTCCGGCAATGAGATCAAAGGGAAGTGTTGGGGAGATGTTACTGACGATTCTATTTCTGAGCCGGATATTGTGGACAGACCGGACGGTCTGGTGTTTTCAAGTTTGGAGATAACTAATCAAAGGGAGAAATTCTTGTGTGATAGCAGTCTTCTTTTGAGTGATGCCCTTTTGCAATATCCAGTTGTGGATGTGACGACCAACGTCCTGACTGGTGATAATATTGTTGACACTTTTTCAGAGTTTTTCTTTTTAGAGAAAAAGAAATTACATGTGGAGTTAGGTAAGATTAATAATGTTGTTGATTTTTATAAATCTTCATTGACCGGGAAGAATGCTTTTTATAATAAGGTTTGGAGCTTGCGAAACAAATTCGACGATTCGTCGTTATATGTTTCGGAAAACTCAAAGGTCTGGTACAAGTTGAAACAGGGTGAGAAGGGTCATGTTCAACTTGAAGGTGTGTGCAAATACACGCTTGACAACAAGTTGGTTCCTTTCACTTACTTTTATGATGACTTTCAGGTCACCAGTGATGAATTGATGGGCATGTTTTCAAACAGGAGATGTTTGGCATTACAGTCAATCACACCGCGTGAGTCCGGGTTTGACCTTAATAGCATGTTGGAGAATGTCACGTTTTTCAATAAACCTCCTGGTGCCGGTAAGACGACCACCATTGTGAGGAATATGGTGAGAGACATCAAAGGTAATGTGAGGTGTTTGGCCCTGACCTGCACAAATGCTGGGAAGAAAGAGATCATACATAAGCTCAGGAAGGAAGGAGTCAACAATGCATTCAGCTTAGTGATGACATATGACTCTTTTCTCATCAATGGTGGTAAGATGGATACTGATATGGTCTATTGTGATGAGATCTTCATGATTCATGCTGGATTGTGGGTGGCCCTATTATCAATGTTGCAATTCAAGAAAATGGAGTGCTACGGTGACAAAAACCAGATTCCATTTATCAATAGAGTGCCGAATACTCTTTGTCAGTACTCCCAGAAGATATTTTTCTTGTTTAGGATGATCCACGACAATGTTTCATACAGATGCCCGCCAGATGTTTGCTACATTTTGTCTAATTTGAGAGATGCTGCCGGTAATCTGTTGTATCCAAATGGTGTCAAGGCTGTGGGGCCGAACAGCAATCTTCTCCGTTCTATGTTCGTGGTGCCACTGAGATCTGCCGAGGAAGTTCCATACAGTCCGGATGTGAAGATGATTGCTTTCACCAAACCTGAGAAGGACGACATAATGCGGCATGGTAGAACAGCGGATGGTAAGACCAATTCAGCTCAGACTGTTAATGAGGTTCAAGGTGGGACTTTTCCCAAAGTCGAGTTGTACAGATTGAGGCAATATGATAATCCCATCTACAATGATGTCAACCAATTCGTGGTTAGTATATCCAGACACACGGAGGTTATGAAATATAGAGTCTTGTCTACGAAAATGCACGACACTGTTGGACAACATATATCATCTTTGGATAAGGTTGCTGATCATATCATAAGGGAGTGTGCATTTAAACAGCAGGTTTAACACTTATCGGTTGACCATTGAAGGTTGTTATATTCCTGACACTTTTTCGAGACCTGCCTCATCTCATTTGATGGCAGTCAACGATTTTATGTCAGTGGTCAACCCGGGATTAGCTTGGATGCAATTTTTGCACAGAACTATATTGTTTGAGTATGGTGATTTTGACATGCCACCTGTTGAGAAGATGGTCCTTGATTTTTCAAAATACAAGCCTTATGTCGCAGGGGAGTTTGTTGTCTCGAAAATTCTTGGCAAAGGTGAGAGGACGAGACCGGACAGTATGAAACAGGGGATAATCTCATTGTCACATAGAAATTTTTCTGCACCGAGAATAAATGAACGTCTGGACGTTTATAAGACTGCTGAACGTTTATGTCAGAATCTCGTTAGATCTTTCGACTTTTCGAGGTTGTATGAGAACTATGATGTGATTCTTCCTGACATGTTCAAAATTGACGATTGGTTGCAAGATAGAGATGGCTCGAAGTTTGGTCGGATAAAAAGGGATATGGACCACAAATTGTTGGTCGAACAGTTTGAGAGCTTAAAATTCATGATCAAAGGGGAGATGAAACCGAAGATGGATATGTCGTCCTATACAGCTTATAATCCACCGGCGAATATCATCTATTATAACCATCTGGTAAGTATGTATTATTCTCCGTTGTTTCTGGAGGTCTTTGATAGGATATCATACTGTCTTAACAAGAAGATAGTTATGTATTCCGGGATGAATCTAGAAACTCTCGGCACCCTGATTGGTTCTAAACTGCAGAAGCCATTGACATCATATCACACTTTGGAGATCGATTTCTCAAAGTTTGATAAGTCTCAAGGTATCCTATTTAAAGTTTATGAGGGGATGATTTACCGGTTTTTCAAGTTTTCCGAGGATTACTATGCCAACATAGAGGCCACTGAATACTTCATAAAGTATCGTGGTAGGTGTGGAATCAGCGGGGAGTTGGGTGCACAAAGGAGAACGGGGTCACCGAACACTTGGTTGTCAAACACATTGGTTACTATGGGTATCATACTCAGTGTTTACGACCTGGATGATATTGATTTATTCTTAGTAAGTGGTGACGACAGTTTGATCTTTTCGAGTAAACCCTTGAAGAATAAAACTGATGAGATAAACAGAGATTTCGGTTTTGAGGCTAAGATGATAGAGAATTCAGTGCCGTATTTTTGCTCCAAATATATCATCAGTGATAGAGGAAAAATCAGAGTCGTTCCTGATCCTGTGAGGTTTTTTGAGAAGTTGTCTGTCCCAATTCGAGTTCAAGATTTTATGAGTGACACTCTCATGCGGGAAAAATTTAGGTCTTATAAGGACTTGATGAAGGACTTTGATTACGACACAACGTGCGTTTTGGTGGATGCTTTGGTGTGTTATAGGTACAATTTACCACCGATGTGTTCATATGCAGCGTTGTGTTATATTCATTGTCTGTGTGCAAATTTTACAACTTTCAGAAGAGTCTATGAGAGCGATTTGACTGTTGTTATTTAGGTCAGTATGGATCTCACTGGTTGCTTGCGTAAGTTTCGACAGTGTGATCGACTTCTTGAGAGGCTGGGTAATGACGTTTCAGAAGTTCATTTAAGAGCGATCTTAATTGATCTTGATGAGTGTTCTGAGTGTTTGATGCTTTGTGAGCAGGAGTATATCAGAGACACGGACTGCCTTATGTCATTTCTGTTGGCGCTGAAACACTATGAGATTAAATTTCACATGGATATGTTGAATATGATTTATGACTTTAAACTGAAAACGTCCCAGTTGATTCAGGATGTTTTTAGAATTAAAGTAATCATCAGAGTGTATCTCGAGTTGTGTGAAATTGATCCACTTTTGGCTATGACTGAGGCTTGTCAAGACATTCTTGAGAGTGGTATTTTGAACATCGGTTTCATCTCTTCGGCCCTCGGGCATGAACCGAACATATTTATCACAATATTGTCGATGGTCGATTTTATAGTCGTCATTGATGATCGACCACTGGTCTTTATCCCTTCAAAAATAAGGTTTGTTGGCGACAAGTTGGGGTCAGGTCATTTTAGGTGGTTTGATAAGTTTTTCTTTGGGAGTGATATATAATCTGGTTAATATTCAAAGGATGGGTGATCGTTTTGCTTGCGATTCTTTGGATAGTATAGCCAGAGATATACACTCTTTGTATATTTTGTTTTTCTATACGTTTCTAGTAGGTGTTTTGTTGACATTCATAATGTCTTGTGTTAGAGGTGTTATAGAAATTTATCGTGTTAATTAGGGAGTTTTTGATAATGTTTCCTTCCTTTGGTTATTTACTTCGTATTTTATAAAATCCAAAAAATATATTATGCTTTTTACATATTGTTCTGTTTAGTTGGTGTGAAAAGTGATCTACCTAAATTACGTGTTATACACGTAGACCTTGGTAGATCTAGTATATAAATAAATAGGTC

>ToCV-2

GAAATACAAGTCCAGGTGTTTCCTGTGGGTACGCGATGAGCCTCCCCCACGTTAATTACCCCACCGTCACTAGGTGGACGTGATTGTGGGCGCTGCCGGCTTCGGTCGTCGGTGTCTGCTTTCATTTTCTAGTGACGCTATGTTGATTAATATTAACATATAACAAAACAAAACAAAAAGAAAATAAAATAGCAGGCCGAGTACCATAAACTTAACTGAACTGCTCGAGTTTTTGCACATGCCTACGGCTGGTATGTTGCAGCCCCTCTTGCACTTTGTTATAGTGTATGTTATGGTCTCGCCCATATCCCCTTACTTTTTCTTTGTGAAATTTAAATGACCAAACAGTGAGTTTGCACGATCAAATTACTATAAACCGGAAAGTTCAGAGTTACTCTGCATTTTCGAAGTCGGCAAGTTGGTACCGACAGTTGATCTATACGAATTCACTATTGACGTTATTTAGAAACGTGTTTTCGTGAATTGTTATTTGTAATAATAAATGTTAAAGGTTGAGTGGTTGAGCATTTACGTCGTCAGATGGAAACTTTCTTATTAACTCATACATGTTAATGGTTTAGTCTTCCTCTCGCGTATTTAGAATCATTCAGTCTAGCACAACAGTCTCATTGTGTTATCGACAATAATTAGCGGTCCAGTTAACTCAGTTTAACTTGATTCCGTTTGTTTTTCCGATTCTTTTATTTGAACTGTATTCTTGTCTTTTGTATTATGAGTATTAAAGCTGGTTTGGATTTTGGTACTACGTTCAGTACTATTAGTTGTTTCTATAATAACAAATTGTTTTCATTAAAACTCAATGGGACCGAGTATATTCCAACTTGTCTCTCCATAACTCCAAATAATGAGGTGATAGTCGGAGGCCCTTCTCAAGTTTTAGAAGCTTCCGAAACTCCGTCTTGTTATTTCTATGATTTGAAAAGATGGGTTGGTGTCACTTCGGTCAATTATGAGGTAGTGAAAGCGAAGATAAACCCAATGTATAAAACGCGTTTATCTAATAATAAAGTGTATATAACTGGTATCAATAAAGGTTTCTCGACCGAGTTTTCGGTTGAGCAACTTATATTACATTATGTTAACACTTTAGTTCGATTGTTCTCAAAAACAGAAAACTTAAAAATAACCGATCTCAACGTGTCTGTTCCGGCTGATTACAAGTCTGGGCAGAGACTTTTCATGCAGGCAGTTTGTTCCTCTTTGGGTTTCAATTTACGTCGCATAGTCAATGAACCGTCGGCTGCCGCTATTTACTGCGTTTCTAAATATCCGCAGTATGCTTATTTCTATATTTACGATTTTGGTGGCGGTACTTTCGACACTTCTTTAATAGTGAGATATGGTAAGTTTGTCACTGTTGCTGATACCCAGGGAGATTCGTTTCTTGGTGGGCGAGATATAGATAAAGCCATATCGAAGTTCATAATGGACAAAAATGCTTTGAACACCCCACTGTCGGCAGATATGTTAGCGTCTATAAAGGAAGAGACAAATTCTACCGGGCGCAGTTCATACAATATAATAAGTGATGATGGGAGTATAATCAATATTCAGTTCACGTTTGACGATTTGGTCAAGTGCGTTGAACCATTCACTAGACGCAGTTTTTCAATACTTCGAAGTCTCGTTTCTCGTAACAAAACTTCGAATGGAGCGCTGTTTCTTGTCGGTGGTTCTTCATTGCTTAGACCGATTCAGAATAGAGCAGATGTTTTTGCGCGTAATCATGGGTTGGCTCTCATTATAGACCCAGATCTCAGAGCTGCCGTGTCATTTGGTTGTTCAATGCTCCATGCACAAGAGGATTCTGGGAATATGACATATATAGACTGCAATTCACATCCGTTGATGGATTTGGGTTTATATTGTCATCCTAGGATTATCATCAGAAAACCCATGTCTGTTCCGTACACACACAAGATTGAGAGGGAGGTCACAAGATTTATCACCACTGCTCTCAATGTTTATGAAGGGTCTGACCTTTTCGTACTTAACAACGACTGGTTAATCAGCGCAGATGTTGACTATTCAAAGTATGCTAAAATGGGTGAGACATTAGTTAGTGTTTATAAGTACACCATTGACGGCATTTTGGAACTGTCGATGGCTAATAAAACCACTGGCAAGTCGTGGGTCCTTCCGAACACCTTTGCCAGGTCCGAGAAGATAGTCATTAGTGATTTAACCTTGACTCAATTGTCGAATGTCGATGAATTGGCCACTATAGTGTCAATTTTGAGTTATTTTGATACAACATTCAACTATCTTACTTCCATGTTCAACACTCCGTCGATTTTTGAGCGAGAAGTTGGAAAGATATCTGACGCCAAAGGTCTTTATAACCGACTCGTTGAACAAAACAGAAATTTCTCTTGAAGGTTTGTTATGTTAGTTTGTTTAGTACTGAGAACTAACTGTCTTGTTAAAGTCTTGTATTTCTCTGATACTCCAATTGTCGAGTCCGGCCCATTAGTAATAAGAAGCTCAGATCCGACCATTATAGAAGACCTTCTAGAGTACTTACCTTTCGTCACTGAGTCATGGAGCCCGTTGATACTTCCGAAAGAGTCAAAAGACTTTTCTCAGTAGTATTCAAGAAATCGAACAACGATGAGAAGATTCATAAATTGGCTGATTACCTCTTGAAATATTATTCAACGGAAAACAGAAATTTGTATAGGACGACCATTAATAATAGAGCTTTCTCTTTTACTTCTACATATTCGGTCTCTGGTGGTAAAGTTTATCTCGACACCAAGGAACCTTGGCAGGTTGTGAAGCTGATCATCATATATTTGTACAAGGTAGAGCCTGGTTATCTTAAGAAAACTAACTACTCTCCTGAAAATCTTTTCGCTAGATTGAGGTTCGATGATTATTACGATGAGTGGAATAAGTATTTTGACAAGGATGTCAACGATTATCTTGCCGACCATCCTGAAGAGGGATGTTTGTACACCATGAACGACATTGTGAAGGAATATCCGGGTGAAGAACCGACTGCACAACTAACTCTATACAGGGTTTGTAACTCGCTTGGAAAAAAGATATCGGTTCGAGAACTTAAAGAGGGAAAAATTAGTGCTTTTAAAATTGAGTCAAAAACTGATAACGCTGAAATTGGAGAAGGTGTTGGTGGTAATGCTCTGTTCAAAGAGTGTGTTGAGACTTTGCAGAGTTATTTGCTCTTGAATTCTTCCAAAGCGGGGCGGGAGAAGATCCGCGCTAATGCTAAGATTTTTGAGTGCTATTTGTCGAGTCTGGTTCCAAAAGGTCTAGATAAGAAATTAGCGGCGAATCCATTGGTTGTGGCTAAATTCGTTAATGCGTTTACGGTCCGGACTGTGAACAGCAAGGGATTTGGTGACAATTTTAAGGCCGTGAAGGAATTGTCTCCTGAAATTTTGAGTTTCATCAAGAGAGTGTTTTTGGTTGACGCCAGGCTTAATGAAGATGTGTTGTTTATAGCACTCCCGAAGAACTCTGTAGTTGAGATTCTTGGTGACAAATTTGCTGTCGGTGAATATTTAAGAGTGCAAAATGTTTTGCCAGCTTCGAGCAACTCAAGTAGCCTCCCACCAGATATCGATAAGTGCGTGTCTGATGCCTTGGTTACTTTCATGCGGTCGTTCGGTAATTTTCAACCTGCCTTCATACTAGATATTTGGTTATTTGTGTTTGGGAAGATGACCACCAATTCCAAACTTTGGAGAGAGGATAATGAGATCCTAGTGACGGTGGGGGACGTAGTTGTGAAATCAACTACCAGTAGGTTATTGTCACATGTGAAAAACTGTGTCAAACGGGACTTTCCCCAGTTCTCGACCGACAATATAATCAGACAGTGGGCTAATTTGAGGGGTGACAGAGCGAAGCAAATGTTTCAATTGATGAACTTCAGACCCGGTTTGTTTTCGAGTATACCGGGTATCAAACCGTATATGCGCTTCGACTTCTTTAAGATGTTAGATTTGTCGAAATGTACTCGTGAAGAAATTGAAAGTTATCAAACATTACGTCGGGTGACGGAAAGTAGGTCCAATAAGACTGCTTGTGACGATAGGTGTTTGGAGTCATGGATCTTGAGGAAATGATCAAAGAGTTAGGTCTGGCTAAAGTTGAGAGATTTCTCACTGTCTATAATCAAGGTAGGTTTGTAGCTTTCGGAAATATAGAAACTCTACTCTGCCTGATTAATCAACATTTTGTGGAGTTTAATCCTCAAAGAGCTAAACTGGACATTGAATTGTCTGAAGTGAGAGATTTCTTGAGGTGTTTTGAATCTTTTAGAAGCTTTGGTTTAAGGAAATAATGGAGAACGATGCTGTTACAAACACTGGTGATAACGGTGGCAGCCGCAATCCTCTGGTTAGACCGTTAGATGATAGCGTAGATGACGAGGTGCAGAACTTAGGCAGGAGGGACGATTCGACATCTCTCATTCCGGCTAATCCTAATCGATCTTCCAGTTGGGCTTTGTTGAACCCGGATACTATTAATTATAACGAGTTAAGGAAATTGAAGGTACACTCCACTAGGGGTGATACTCTTACCTTGACTCAGGAAGAGGAGTTCGAGAAGATACTCGAATCCTTCTGCAGGCGAATAATCGGTGAGACCCCGATGACAGATAAGATTTTCGCTGGTTTCTACATGTCTATGTGTCAGGCCATTGTAAACCAAGGGACCTCAGTTAAAGCAGCCGGTAATAACAGTCTTGAAAATTACTTTGAGGTAGATGGCGCGAGATTTAAGTGGAAAACTCCGGATTTGATAAATGAGGTTAGACCCAAAATGTCCGATGTTCCAAACGCTATACGTCGGTACGCCAGGAGTCATGAAAAGATTATTCAGGATTTTATTAACTCCGGTCTTATTAAGCCTGATTATCATTTACAATTCAAACATGGCGTATTACCAAGCCATGTGTTTGGTACCGGCGATTATATAAATGGTTCGTTGATGAATATCTCAGATGATCAACTTATCTCGAACCTGCTTATGAAAAGAAACGCTTTGTGCAAGGGTAACGAGGGCAAGGAACTGTACAACGTTAACCAGCTTGCATCGATAACCGGTTGCTAAATTATATGGATGAAAATGAAATCTATGAGGATCAAGAGGATCTCTCTGCTCGTGGCGGTGGGGGTTTCTATTACCAGACCGTGACTTTGGGTTCCGGTGATGTGTTTCCCGTTGATTTAGCCCTAACGAGATCGGCTGAATTTGATTCGACAATTTTCTCCTTATATATTAGGTTCGTAATTAAGGAGGGGAATGTGCGTTTAAAGATCGATTTTGGAAATAATTGGGATGTGTCTATGCAGCAGGTGAGACTTTCTGGATGGTTTGCGGCGTTTGGTAAAATTGAAAAACCGAGAACCGCCAGGTCTGGATGGTCATACCCAATAAAATTGTTTAAAGAGGCTGGAGAAGTCATAGTGTCCATTAGTGGTTGGAGGTGTTATAAAATTTATAATGGATATCCTGTAGATCGCGTTGATTTGGTTCTGGCAGTACCCGTTCGTGAAGTAACAGCCGATTTAAAACGACCGTTGGTTGGGGATTACGTCAACTTTCATGATGTATTTACTCTTATAAAGAGTAAAAATATTGACATCACTTTACCTAACCCGAGTCTGATATTCAACGATTCGACAAGTAAGGTTAATTTAGATGTGTCTCCAGGTGCGCGAAAACAAATTGCTCAAGTTAAGGCTGAGAAAGACTTGAACATTAAGAATCCTGAAGACTTAAAGCCTGATGTTCCTAATGATTCGCTGAGTGAAGTCGAATATCATAATCACTCTGATGTTTCCAGTGTTTTCAGATTGTATTACACATGGAGGGTTGAAAGAGATTTTGAAAGATCAGTTGAGTCGAGAATTTTCTTTCCGAATATATTTCCGACCGATTTCACAATACTTCAACAAATGTGGTACGGGACGACTGCCGGTAACGTTGAGACTTTTGTGGAGATAGGTAAAAATGATAGGAAGTTCAACGTTGGGGTCGCCGCTTGGAAGGACAATTCATTTGGACATTTCAAATTAGATGGCCCGACTTTAGCGAAGATCTCAACAATTCCAGGCAGGTTCGTGGACCATAAAATTGAAAAAGACTCTAAAGGACATTTGATTGTGAGTATTGATAATACTGTTCTTGTGCGTACTAACAAACCGATAGTCAAACCGAGTATTCAGATCGGCTGGGAATTTCATTTACCATGGGACGCGATCAGAAAGTATGGAGTTGGTAATTTGGCCAGGTTCACAGACATCATCAAACCCAATTACATCAAGTTTGATAGTTCTGAAGTTCCTTTGGTACAAACTAATACTATAGAAAGTGATCGTTCCAAGTCTGGTCATAAACTATCTCTAGTCAACCTGAAAAGTTTCAGACGTATTAGTTCTACTGCAGATTTCTTCTTCGAACCACCACCACCCTCTGAGTCCGATGACAAAAGTTGGGAAGATAAGGCCCAAACCGAAGTGGATATAAAGAAAGAAGAGACTATTCCGCCTAATGAAGGCACCTCTTCCTCTGATTTGCCGAGTGAGAAGTCACAATTTGTTGCAGCTAATCATTACCTCTTGTCGATAGTTGAAGACAGGAATATTTTTAAAGCGGCTGTAGATCGGTATACCGGCTTGGGTTTCTCAAAGGATCAAGCTGTGTTGATAATATATCAATTGGGGGTAACATTCGGCACTTCCAGAAATTGTTGCAGTGATAATTCATCGTTTCTAGTCTGGAAGACTGATACCGGAGCGCAGGTTATAATCAGAAAGGGCGCACACTCCAGGTTTCTCAATTCACTGGTTAAATATCCTTGTAACGTGGAGAGATTGATACTACGAAGACGTAGTGCGGAGATATTGGCGTTGTTGAGGAACAAGAAATTGGCTTACCCAGATAGATTGGCCAAAAAGAAAGGGGTAAGTCAGGGATTCACATATATGGCATGTGATTTTCTCGATTACACTGCGGTAACGTTAACTCAAGAAGAGCAGTTGACTATGAATTCTGTTGTGCAGTACGTGAGACTCCATAATAAACATCGAAGAAGCATTGTGAGCACGAGTCAGCTTTTCTGATCGATGGAGGTCGTGTACAATTCAGACGATGTTAACAGTGGAACTGGATCCGGTGAAGATGTAAATACGACCGTGGCCAAGAACTTTTATACTATAACTCACGTTATGAGTAACTACCGTAATTACACACCAGACGAAATTAAGGATGCTGTGAATGTAGGTTATGGGTTACTGAATTTGTGTGAGAGGTTGGATAGAGATGTAATACTTGTGTCTCCGAATTCACCAGTTTACAACAATTACCGAGATGCCGGAATTCCACACAATTTACTTATGGAAAATACTGCACGGTATTTCCCAGTAGTTAATCCGAGTGAATTAGGAAAAATTCTATTGGGTCATATCAGTGTTTTAAAGTTTTTGGAGTGTTTCACAAGATACGGGGTTGACGATATGCTAATTACAAGATTGTTCTCAAATTATGTCTTGTGGTCCACTGGTGACGTGAACGCAGCATTATATTCTATTTATCAACAGGATTTTCACTTTCCTGTTGAGGTAAGAGCGAATTTTAATTTCTTATTTTTGAATTCAAGTGAAATTGACAGAAGGTTGAGTAACATTAGAAGGAAAGGTTATCCAAACTCTGAGAATTTCAATTGGTTCAAAAATATGATAAGTAATTACTTATATTTTGATTTTGTGTTCAGATACTCTGGTACAAAAATCAATATAGAAAGAATCTCAAATTACTATATTTGATTTTCAATATGATTTCCACTTATTTTACCTTAATAGGTTTAATTTTCTTGGTGGTATTCTGTTTTGTTTTATTATGTTATTTCGTCTTCACTGTCATTAAATTCTTCGCGAAAGATAAGATGAGTGACGATGATTGTCCTTATGTCAATAATGTTGCTCCATTCGGGAGTAACAGGTTTAACTCACAACCTCCAATCGTTCGTTAAAGTACTATATTACGGTTGGATTAATAAAAATTTATAAGGAGTTTTTGATAAAGTTTTCTTCTTCGGGTTATTTACTTCGTATTTTATAAAATCCCAAAAATATATGAAAGATTTTACATATTGTTCTGTTTAGTTGGTGTGAAATTCTATCTACCTAAATTACGTGTTATACACGTAAACCTTGGTAGATTTAGTATATAAATAAATAGGTC

>TYLCV

TAATATTACCGGATGGCCGCGCCTTTTCCTTTTATGTGGTCCCCACGAGGGTTCCACAGACGTCACTGTCAACCAATCAAAATGCATACTCAAACGTCAGATAAGTGTTCATTTGTCTTTATATACTTGGTCCCCAAGTATTTTGTCTTGCAATATGTGGGACCCACTTCTAAATGAATTTCCTGAATCTGTTCACGGATTTCGTTGTATGTTAGCTATTAAATATTTGCAGTCCGTTGAGGAAACTTACGAGCCCAATACATTGGGCCACGATTTAATTAGGGATCTTATATCTGTTGTAAGGGCCCGTGACTATGTCGAAGCGACCAGGCGATATAATCATTTCCACGCCCGTCTCGAAGGTTCGCCGAAGGCTGAACTTCGACAGCCCATACAGCAGCCGTGCTGCTGTCCCCATTGTCCAAGGCACAAACAAGCGACGATCATGGACGTACAGGCCCATGTACCGAAAGCCCAGAATATACAGAATGTATCGAAGCCCTGATGTTCCCCGTGGATGTGAAGGCCCATGTAAAGTCCAGTCTTATGAGCAACGGGATGATATTAAGCACACTGGTATTGTTCGTTGTGTTAGTGATGTTACTCGTGGATCTGGAATTACTCACAGAGTGGGTAAGAGGTTCTGTGTTAAATCGATATATTTTTTAGGTAAAGTCTGGATGGATGAAAATATCAAGAAGCAGAATCACACTAATCAGGTCATGTTCTTTTTGGTCCGTGATAGAAGGCCCTATGGAAGCAGCCCAATGGATTTTGGACAGGTTTTTAATATGTTCGATAATGAGCCCAGTACCGCAACCGTGAAGAATGATTTGCGTGATAGGTTTCAAGTGATGAGAAAATTTCATGTAACAGTTATTGGTGGGCCCTCTGGAATGAAGGAACAGGCATTAGTTAAGAGATTTTTTAAAATTAACAGTCATGTAACTTATAATCATCAGGAGGCAGCCAAGTATGAGAACCATACTGAAAACGCCTTGTTATTGTATATGGCATGTACGCATGCCTCTAATCCAGTATATGCAACTATGAAAATACGCATATATTTCTATGATTCAATATCAAATTAATAAAATTTATATTTTATATCATGCGTTTCTGTTACATTTATTGTGTTTTCAAGTACATCATACAATACATGATCAACTGCTCTGATTACATTGTTAATGGAAATTACACCAAGACTATCTAAATTCTTAAGAACTCCATATCTAAATACTCTTAAGAAATGACCAGTCTGAGGCTGTAATGTCGTCCAAATTCGGAAGTTGAGAAAACATTTGTGAATCCCCATTACCTTCTTGATGTTGTGGTTGAATCTTATCTGAATGGAAATGATGTCGTGGTTCATTAGAAATGGCCGCTGGCTGTGTTCTGTTATCTTGAAATAGAGGGGATTGTTTATCTCCCAAATAAAAACGCCATTCTCTGCCTGAGGAGCAGTGATGAGTTCCCCTGTGCGTGAATCCATGATTGTTGCAGTTGAGGTGGAGGTAGTATGAGCAGCCACAGTCTAGGTCTACACGCTTACGCCTTATTGGTTTCTTCTTGGCTATCTTGTGTTGGACCTTGATTGATACTTGCGAACAGTGGCTCGTAGAGGGTGACGAAGGTTGCATTCTTGAGCGCCCAATTTTTCAATGATATATTTTTTTCTTCGTCTAGATATTCCCTATATGAGGAGGTAGGTCCTGGATTGCAGAGGAAGATAGTGGGAATTCCCCCTTTAATTTGAATGGGCTTTCCGTACTTTGGGTTGCTTTGCCAGTCCCTTTGGGCCCCCATGAATTCCTTGAAGTGCTTTAAATAATGCGGGTCTACGTCATCAATGACGTTGTACCACGCATCATTACTGTACACCTTTGGGCTTAGGTCTAGATGTCCACATAAATAATTATGTGGGCCTAGAGACCTGGCCCACATTGTTTTGCCTGTTCTGCTATCACCCTCAATTACAATACTTATGGGTCTCCATGGCCGCGCAGCGGAAGACACGACGTTCTCAGCGACCCACTCTTCAAGTTCATCTGGAACTTGATTAAAAGAAGAAGAAAGAAATGGAGAAACATAAACTTCTAAAGGAGGACTAAAAATCCTATCTAAATTTGAACTTAAATTATGAAATTGTAAAATATAGTCCTTTGGGGCCTTCTCTTTTAATATATTGAGGGCCTCGGAT

Supplementary data 3: Programs and commands used for get cumulative yield table

#get_cumulative_yield_table

#install NanoComp

#python get_cumulative_yield_table.py summary1.txt summary2.txt summary3.txt > my_table_of_cumulative_yields.tsv

import nanoget

from argparse import ArgumentParser

from nanoplotter.timeplots import check_valid_time_and_sort

def main():

args = get_args()

dfs = check_valid_time_and_sort(

nanoget.get_input(

source="summary",

files=args.summaries,

threads=8,

readtype="1D",

names=None,

barcoded=False,

combine="track"),

timescol="start_time"

).set_index("start_time")

cumsums = [get_cumsum(dfs, dataset) for dataset in dfs["dataset"].unique()]

print(

cumsums[0].join(cumsums[1:], how="outer").to_csv(sep="\t", na_rep='NaN')

)

def get_cumsum(df, dataset):

return df.loc[df["dataset"] == dataset, "lengths"] \

.cumsum() \

.resample('10T')\

.max() \

.divide(1e9) \

.to_frame() \

.rename(columns={"lengths": dataset})

def get_args():

parser = ArgumentParser(description="Extract cumulative yield information to table.")

parser.add_argument("summaries",

help="(compressed) summary files generated by albacore or guppy.",

nargs='+')

return parser.parse_args()

if __name__ == '__main__':

main()

Supplementary data 4: Estimations of costs of sequencing per sample using nanopore and Illumina sequencing approaches (price for Illumina sequencing is taken from Vasquez Iglesias et al., 2022).

|  | MinION cDNA-PCR (with ribosomal RNA depletion step) | MinION cDNA-PCR barcoding (with ribosomal RNA depletion step) | MinION cDNA-PCR barcoding* (with ribosomal RNA depletion step) | MiSeq barcoding (including ribosomal RNA depletion step) |
| --- | --- | --- | --- | --- |
| Number of samples | 1 | 12 | 24 | 24 |
| Flowcell R9.4.1 | 810 € | 70 € | 34 € | / |
| Oxford Nanopore Technologies  library preparation kit | 90 € | 20 € | 9 € |  |
| Other consumables | 30 € | 30 € | 30 € |  |
| Ribodepletion kit | 95 € | 95 € | 95 € |  |
| Cost per sample | 1,025 € | 215 € | 170 € | 189 € |

*This is an Early Access product (March, 2022)

# Supplementary Tables

Supplementary table 1: Sample properties used for MinION sequencing followed by general sequencing outputs: Number of raw reads and nucleotides, Mean read quality, Trimming paramethers, Number of trimmed reads and nucleotides, Average trimmed reads length

| **Sample Number** | **Plant** | **Type of material** | **RNA isolation type** | **MinION Sequencing type** | **MinION flowcell name** | **Number of active pores** | **RNA loading input into the flowcell (ng/ul)** | **Number of raw reads** | **Number of raw nucleotides** | **Mean quality score** | **Trimming parameters** | **Number of reads after trimming** | **Number of nucleotides after trimming** | **Average trimmed reads length** |
| --- | --- | --- | --- | --- | --- | --- | --- | --- | --- | --- | --- | --- | --- | --- |
| I | Solanum lycopersicum | frozen (-80 °C) leaf | RNeasy | directRNA sequencing of totRNA | fak04958 | 1253 | 0.877 | 1,085,202 | 1,073,653,862 | 10.1 | --headcrop 40 --tailcrop 50 -l 100 | 1,005,601 | 973,270,059 | 968 |
| I | Solanum lycopersicum | frozen (-80 °C) leaf | RNeasy | directRNA sequencing of ribosomal RNA-depleted total RNA | fah67285 | 1310 | 0.833 | 1,230,755 | 444,669,627 | 7.7 | --headcrop 50 --tailcrop 50 -l 100 | 631,545 | 302,579,594 | 479 |
| I | Solanum lycopersicum | frozen (-80 °C) leaf | RNeasy | cDNA-PCR sequencing of totRNA | fah67150 | 1233 | 0.994 | 198,916 | 190,112,771 | 7.8 | --headcrop 50 --tailcrop 40 -l 100 | 174,753 | 170,495,413 | 976 |
| I | Solanum lycopersicum | frozen (-80 °C) leaf | RNeasy | cDNA-PCR sequencing of ribosomal RNA-depleted total RNA | fak29439 | 1486 | 3.92 | 578,725 | 233,045,588 | 6.7 | --headcrop 50 --tailcrop 30 -l 100 | 349,320 | 179,805,033 | 515 |
| II | Brassica oleracea | lyophilised leaf | RNeasy | directRNA sequencing of totRNA | fak04840 | 1204 | 1.69 | 1,569,963 | 825,695,249 | 8.1 | --headcrop 40 --tailcrop 50 -l 100 | 1,102,915 | 672,453,469 | 610 |
| III | Nicotiana tabacum | fresh leaf | RNeasy | directRNA sequencing of totRNA | fak05019 | 1376 | 1.45 | 2,657,455 | 1,253,113,279 | 8.3 | --headcrop 40 --tailcrop 60 -l 100 | 1,926,424 | 958,706,417 | 498 |
| IV | Solanum lycopersicum | dry seeds | CTAB + RNeasy | directRNA sequencing of totRNA | fah75033 | 824 | 0.707 | 1,242,419 | 503,054,840 | 8 | --headcrop 40 --tailcrop 60 -l 100 | 929,268 | 365,524,270 | 393 |
| V | Phaseolus vulgaris | fresh leaf | RNeasy | directRNA sequencing of totRNA | fah65119 | 1320 | 3.14 | 1,691,903 | 1,814,273,701 | 8.7 | --headcrop 40 --tailcrop 60 -l 100 | 1,429,664 | 1,635,982,729 | 1144 |
| Neg.control | Nicotiana tabacum | fresh leaf | RNeasy | directRNA sequencing of totRNA | fak04973 | 1270 | 5.04 | 2,139,828 | 2,003,456,513 | 8.5 | --headcrop 40 --tailcrop 60 -l 100 | 1,774,094 | 1,775,098,051 | 1001 |

Supplementary table 2: Sample properties used for Illumina rRNA-depleted totRNA sequencing followed by general sequencing outputs: Number of raw reads and nucleotides, Number of trimmed reads and nucleotides and Average trimmed reads length

| **Sample Number** | **Plant** | **Type of material** | **RNA isolation type** | **Number of raw reads** | **Number of raw nucleotides** | **Number of reads after trimming** | **Number of nucleotides after trimming** | **Average trimmed reads length** |
| --- | --- | --- | --- | --- | --- | --- | --- | --- |
| I | Solanum lycopersicum | frozen (-80 °C) leaf | RNeasy | 1,176,594 | 228,469,692 | 1,130,226 | 212,249,557 | 188 |
| II | Brassica oleracea | lyophilised leaf | RNeasy | 7,468,834 | 1,624,123,999 | 7,181,782 | 1,514,646,641 | 211 |
| III | Nicotiana tabacum | fresh leaf | RNeasy | 8,143,718 | 1,692,882,100 | 7,830,500 | 1,577,244,796 | 201 |
| IV | Solanum lycopersicum | dry seeds | CTAB + RNeasy | 8,828,148 | 1,706,612,568 | 8,468,410 | 1,574,397,106 | 186 |
| V | Phaseolus vulgaris | fresh leaf | RNeasy | 6,505,012 | 1,526,818,146 | 6,144,582 | 1,397,798,959 | 227 |
| Neg.control | Nicotiana tabacum | fresh leaf | RNeasy | 4,468,118 | 1,047,402,755 | 4,272,178 | 971,255,020 | 227 |

Supplementary table 3: Parameters used in CLC Genomic Workbench 12, 21 for mapping reads to viral/viroid genomes.

| **Mapping reads** | Diagnostic workflow | All reads and Subsamples for comparison analysis |
| --- | --- | --- |
| Masking mode | No masking | No masking |
| Masking track |  |  |
| Match score | 1 | 1 |
| Mismatch cost | 1 | 1 |
| Cost of insertions and deletions | Linear gap cost | Linear gap cost |
| Insertion cost | 2 | 2 |
| Deletion cost | 2 | 2 |
| Insertion open cost | 6 | 6 |
| Insertion extend cost | 1 | 1 |
| Deletion open cost | 6 | 6 |
| Deletion extend cost | 1 | 1 |
| Length fraction | 0.8 | 0.95 |
| Similarity fraction | 0.8 | 0.95 |
| Global alignment | false | false |
| Auto-detect paired distances | false | true |
| Non-specific match handling | Map randomly | Map randomly |

Supplementary table 4: Parameters used in CLC Genomic Workbench 12, 21 for de novo assembly.

| **De Novo Assembly** | Diagnostic workflow | All reads and Subsamples for comparison analysis |
| --- | --- | --- |
| Mapping mode | Create simple contig sequences (fast) | Create simple contig sequences (fast) |
| Update contigs | true | true |
| Mismatch cost | 2 | 2 |
| Insertion cost | 3 | 3 |
| Deletion cost | 3 | 3 |
| Length fraction | 0.5 | 0.5 |
| Similarity fraction | 0.9 | 0.8 |
| Alignment mode | local | local |
| Match mode | random | random |
| Create list of un-mapped reads | false | false |
| Automatic bubble size | true | true |
| Bubble size | 50 | 50 |
| Automatic word size | true | true |
| Word size | 20 | 20 |
| Minimum contig length | 100 | 200 |
| Guidance only reads |  |  |
| Perform scaffolding | true | true |
| Auto-detect paired distances | true | true |
| Create report | false | true |

Supplementary table 5: Parameters used in CLC Genomic Workbench 12, 21 for mapping reads to viral/viroid genomes. *parameter used for viroid

| **Mapping contigs** | Diagnostic workflow | All reads and Subsamples for comparison analysis |
| --- | --- | --- |
| Masking mode | No masking | No masking |
| Masking track |  |  |
| Match score | 1 | 1 |
| Mismatch cost | 1 | 1 |
| Cost of insertions and deletions | Linear gap cost | Linear gap cost |
| Insertion cost | 2 | 2 |
| Deletion cost | 2 | 2 |
| Insertion open cost | 6 | 6 |
| Insertion extend cost | 1 | 1 |
| Deletion open cost | 6 | 6 |
| Deletion extend cost | 1 | 1 |
| Length fraction | 0.7 | 0.9/0.5* |
| Similarity fraction | 0.7 | 0.9 |
| Global alignment | false | false |
| Auto-detect paired distances | false | true |
| Non-specific match handling | Map randomly | Map randomly |

Supplementary table 6: Parameters used in CLC Genomic Workbench 12 for Pfam domain search

| **Translate to Protein** | Diagnostic workflow |
| --- | --- |
| Genetic code | 1 Standard |
| Translate CDS | false |
| Extract existing translations from annotation | false |
| Translate ORF | false |
| Extract existing translations from annotation | false |
| Reading frame +1 | true |
| Reading frame +2 | true |
| Reading frame +3 | true |
| Reading frame -1 | true |
| Reading frame -2 | true |
| Reading frame -3 | true |
| **Pfam Domain Search** |  |
| Database | Pfam-A v32 |
| Use profile's gathering cutoffs | true |
| Significance cutoff | 1 |
| Remove overlapping matches from the same clan | true |

Supplementary table 7: Datasets of subsamples for each sample/sequencing type combination

|  | Sample I | | | | | Sample II | | Sample III | | Sample IV | | Sample V | |
| --- | --- | --- | --- | --- | --- | --- | --- | --- | --- | --- | --- | --- | --- |
| Sequencing type | MinION directRNA sequencing of rRNA-depleted totRNA | MinION cDNA-PCR sequencing of rRNA-depleted totRNA | MinION directRNA sequencing of totRNA | MinION cDNA-PCR sequencing of totRNA | Illumina rRNA-depleted totRNA sequencing | MinION directRNA sequencing of totRNA | Illumina rRNA-depleted totRNA sequencing | MinION directRNA sequencing of totRNA | Illumina rRNA-depleted totRNA sequencing | MinION directRNA sequencing of totRNA | Illumina rRNA-depleted totRNA sequencing | MinION directRNA sequencing of totRNA | Illumina rRNA-depleted totRNA sequencing |
| Datasets of subsamples in millions | / | / | / | / | / | / | 1500 | / | 1500 | / | 1500 | 1500 | / |
|  | / | / | / | / | / | / | 1300 | / | 1300 | / | 1300 | 1300 | 1300 |
|  | / | / | / | / | / | / | 1100 | / | 1100 | / | 1100 | 1100 | 1100 |
|  | / | / | 900 | / | / | / | 900 | 900 | 900 | / | 900 | 900 | 900 |
|  | / | / | 700 | / | / | / | 700 | 700 | 700 | / | 700 | 700 | 700 |
|  | / | / | 500 | / | / | 500 | 500 | 500 | 500 | / | 500 | 500 | 500 |
|  | 300 | / | 300 | / | / | 300 | 300 | 300 | 300 | 300 | 300 | 300 | 300 |
|  | 200 | / | 200 | / | 200 | 200 | 200 | 200 | 200 | 200 | 200 | 200 | 200 |
|  | 100 | 100 | 100 | 100 | 100 | 100 | 100 | 100 | 100 | 100 | 100 | 100 | 100 |
|  | 50 | 50 | 50 | 50 | 50 | 50 | 50 | 50 | 50 | 50 | 50 | 50 | 50 |
|  | 30 | 30 | 30 | 30 | 30 | 30 | 30 | 30 | 30 | 30 | 30 | 30 | 30 |
|  | 10 | 10 | 10 | 10 | 10 | 10 | 10 | 10 | 10 | 10 | 10 | 10 | 10 |

Supplementary table 8: cumulative yields of reads in gigabases for every 10 minutes of sequencing from MinION basecalling summary.txt files

start_time /DATA/Sample-III/sequencing_summary.txt /DATA/Sample-IV/sequencing_summary.txt /DATA/Sample-II/sequencing_summary.txt /DATA/Sample-I-cDNA-PCR-rRNA/sequencing_summary.txt /DATA/Sample-I-directRNA/sequencing_summary.txt /DATA/Sample-I-directRNA-rRNA/sequencing_summary.txt /DATA/Sample-I-cDNA-PCR/sequencing_summary.txt

0 days 00:00:00 0.010860549 0.006463064 0.0048842 0.006058803 0.006311501 0.00856043 0.004163318 0.005928074

0 days 00:10:00 0.023547775 0.014272866 0.010781017 0.013604439 0.012425353 0.020136867 0.009304021 0.01156716

0 days 00:20:00 0.036363033 0.022611055 0.016888291 0.02151583 0.017237041 0.032722579 0.014444421 0.016663011

0 days 00:30:00 0.049246463 0.031099664 0.023058942 0.029490285 0.021450571 0.045409335 0.019635422 0.021556681

0 days 00:40:00 0.06228675 0.039914439 0.029185716 0.037584226 0.025199463 0.058336857 0.024892599 0.026152221

0 days 00:50:00 0.075521727 0.048809603 0.035324593 0.045688388 0.028668289 0.071117448 0.030193081 0.030313956

0 days 01:00:00 0.088721503 0.057858024 0.041579699 0.054038454 0.031597153 0.083761126 0.035309977 0.034279845

0 days 01:10:00 0.101866087 0.066927419 0.047896151 0.062436732 0.034288199 0.096331749 0.040368899 0.038254266

0 days 01:20:00 0.115081337 0.076059687 0.054197437 0.070674377 0.036772377 0.108825033 0.045367337 0.041954724

0 days 01:30:00 0.126341721 0.083255352 0.059310198 0.076611735 0.039048721 0.11956084 0.049730977 0.045105971

0 days 01:40:00 0.13943363 0.09174912 0.065323741 0.083905565 0.041370106 0.132398326 0.055028965 0.048594548

0 days 01:50:00 0.152641055 0.100359218 0.071340606 0.091555415 0.043507757 0.145233471 0.060189068 0.051704374

0 days 02:00:00 0.16587894 0.109120285 0.077385416 0.099459559 0.045731579 0.157944786 0.065293287 0.054621146

0 days 02:10:00 0.178992503 0.117917473 0.083424653 0.107481433 0.048019107 0.170460152 0.070262773 0.057395804

0 days 02:20:00 0.192243302 0.126853824 0.089310347 0.115372101 0.050289841 0.182806678 0.07509914 0.060073185

0 days 02:30:00 0.205221551 0.135784684 0.095159723 0.123275133 0.052482496 0.195218658 0.07984628 0.062684304

0 days 02:40:00 0.218423183 0.144708813 0.101050245 0.131083857 0.054593804 0.207420593 0.0845109 0.065071038

0 days 02:50:00 0.231746595 0.153697987 0.106827439 0.138881586 0.056690175 0.219394918 0.089293104 0.067342537

0 days 03:00:00 0.242705488 0.16133009 0.11158598 0.144464967 0.058457581 0.229146388 0.093221615 0.069211554

0 days 03:10:00 0.255330283 0.170194944 0.117209408 0.151171901 0.06043701 0.24082108 0.098028373 0.071830895

0 days 03:20:00 0.268114911 0.179262154 0.122968432 0.158338881 0.062549961 0.252468329 0.102758032 0.073815103

0 days 03:30:00 0.28114465 0.188400012 0.128692081 0.165679702 0.06447223 0.263918523 0.10724121 0.075733626

0 days 03:40:00 0.294177269 0.197478036 0.134454528 0.173003295 0.066365921 0.275191055 0.111620135 0.077865332

0 days 03:50:00 0.30713967 0.206498098 0.140211035 0.180347473 0.068124477 0.286317187 0.116058038 0.080178245

0 days 04:00:00 0.320118176 0.215614631 0.145941716 0.187850004 0.069897281 0.297367406 0.1203879 0.08234325

0 days 04:10:00 0.332931363 0.224636971 0.151537771 0.195236396 0.071739167 0.308083119 0.124681598 0.084291354

0 days 04:20:00 0.3458042 0.233636244 0.157180779 0.202595921 0.073523855 0.31876349 0.128957674 0.086299974

0 days 04:30:00 0.356606396 0.241158102 0.161720419 0.208057014 0.075150095 0.327563175 0.132447153 0.087933214

0 days 04:40:00 0.369349915 0.249928299 0.167034758 0.213831005 0.077154273 0.338211693 0.136621436 0.089876564

0 days 04:50:00 0.382055041 0.258736812 0.172300137 0.22041914 0.079051806 0.34874703 0.140836442 0.091940975

0 days 05:00:00 0.394844389 0.267702359 0.17761644 0.227487675 0.080901842 0.359177929 0.144997151 0.09370887

0 days 05:10:00 0.407722781 0.276859953 0.18266282 0.234672369 0.082713472 0.369290707 0.149128779 0.095484589

0 days 05:20:00 0.420551882 0.285970262 0.187745869 0.241840532 0.084602892 0.37930216 0.153139703 0.097193974

0 days 05:30:00 0.433226811 0.294977601 0.192721902 0.249071838 0.086356458 0.38938483 0.157186599 0.09894664

0 days 05:40:00 0.44584677 0.303918849 0.197717236 0.25615755 0.088210042 0.399219245 0.161065764 0.100643617

0 days 05:50:00 0.458467108 0.312716636 0.202628241 0.263277045 0.090105342 0.40891233 0.164901345 0.102399805

0 days 06:00:00 0.469184101 0.320238628 0.206724735 0.268800185 0.091641579 0.416972393 0.168086908 0.103904726

0 days 06:10:00 0.481645716 0.328994042 0.211795649 0.274609508 0.093284718 0.426385525 0.171870561 0.105656136

0 days 06:20:00 0.494190507 0.337703011 0.216802912 0.280916123 0.095064508 0.435728908 0.175549007 0.107245671

0 days 06:30:00 0.506717213 0.346279471 0.221847593 0.287522462 0.096808939 0.444899777 0.17920999 0.108919602

0 days 06:40:00 0.519199059 0.354914549 0.226870113 0.294211174 0.099065781 0.454009929 0.182891848 0.110311918

0 days 06:50:00 0.531774244 0.363620161 0.231765005 0.300922387 0.100767775 0.462987393 0.186508884 0.11177902

0 days 07:00:00 0.544252985 0.372368718 0.236625635 0.307605899 0.102396001 0.471962141 0.190018247 0.113385588

0 days 07:10:00 0.556654716 0.380976573 0.241414349 0.31421954 0.104029674 0.480843791 0.193458999 0.114932959

0 days 07:20:00 0.569112952 0.389621709 0.246178946 0.320815653 0.105737008 0.489549458 0.196835483 0.116361453

0 days 07:30:00 0.580223943 0.397824413 0.250346661 0.32679052 0.107098994 0.49776361 0.199776661 0.117646244

0 days 07:40:00 0.591714697 0.405401636 0.254536634 0.33141536 0.108887961 0.505722939 0.202992271 0.119148084

0 days 07:50:00 0.603529518 0.413825048 0.258986847 0.336906692 0.110577823 0.514679506 0.206290969 0.120758687

0 days 08:00:00 0.615282491 0.422238939 0.263326783 0.34274275 0.11234307 0.523374794 0.209575018 0.12220826

0 days 08:10:00 0.626958959 0.430767045 0.26767822 0.348841228 0.113987595 0.531817147 0.212772793 0.123538628

0 days 08:20:00 0.638803241 0.439255714 0.271933142 0.354988641 0.11560507 0.540242856 0.215897453 0.124952537

0 days 08:30:00 0.650552968 0.447791333 0.276276347 0.361067723 0.11718598 0.548770447 0.219060674 0.126222601

0 days 08:40:00 0.662246909 0.456281425 0.280499476 0.3671707 0.118683784 0.557338813 0.222206135 0.127330106

0 days 08:50:00 0.673920316 0.46480767 0.284633118 0.373265059 0.120254134 0.565713171 0.225380622 0.128574841

0 days 09:00:00 0.685506029 0.473198021 0.288777072 0.379191213 0.121743214 0.574104457 0.228464675 0.129794659

0 days 09:10:00 0.695614765 0.480309937 0.292263357 0.38376822 0.123319002 0.580831569 0.231069721 0.131034817

0 days 09:20:00 0.70751674 0.488701768 0.296383858 0.389913401 0.124923734 0.588813319 0.234194447 0.132356759

0 days 09:30:00 0.719450752 0.497120124 0.300424721 0.396031135 0.126616309 0.596743506 0.237330849 0.133600464

0 days 09:40:00 0.731371659 0.505462367 0.304436126 0.402151685 0.128106409 0.604674459 0.240339663 0.134789306

0 days 09:50:00 0.743225383 0.513749696 0.308357539 0.408276236 0.129706636 0.612588424 0.243429939 0.135881522

0 days 10:00:00 0.755012398 0.522042825 0.312298691 0.414340864 0.131306431 0.620359377 0.246464948 0.136906131

0 days 10:10:00 0.766736846 0.530225613 0.316226977 0.420363963 0.132843458 0.627992637 0.249468909 0.138134306

0 days 10:20:00 0.778377276 0.538393736 0.319990775 0.42633492 0.134243868 0.635555569 0.252463168 0.139358799

0 days 10:30:00 0.789921213 0.546580229 0.323724319 0.432205606 0.13568668 0.642894013 0.255347314 0.140552429

0 days 10:40:00 0.799489741 0.553381191 0.326898449 0.436507269 0.137109393 0.649249331 0.25788562 0.141729866

0 days 10:50:00 0.810643268 0.561360742 0.330661429 0.441973994 0.138600108 0.656904583 0.260901385 0.142853805

0 days 11:00:00 0.82164271 0.569452188 0.334401296 0.447664726 0.140008075 0.664535384 0.263816117 0.144013932

0 days 11:10:00 0.832602611 0.57769424 0.338091861 0.453314537 0.141450895 0.672039133 0.266719623 0.14508395

0 days 11:20:00 0.843491889 0.585710622 0.341600261 0.458973606 0.142924064 0.679429239 0.269515509 0.146176535

0 days 11:30:00 0.854275641 0.593659807 0.345164491 0.464722888 0.144273543 0.686912981 0.272323855 0.147158492

0 days 11:40:00 0.86510785 0.601623728 0.348644106 0.470418982 0.145817253 0.694220355 0.275126947 0.148278795

0 days 11:50:00 0.875941137 0.609619919 0.352170303 0.475970624 0.14711359 0.701387668 0.277959707 0.149254983

0 days 12:00:00 0.886612175 0.617510666 0.355560107 0.481498879 0.148479695 0.708525407 0.280626631 0.150258087

0 days 12:10:00 0.895738461 0.624213588 0.358382136 0.485745254 0.149815848 0.714440685 0.282983845 0.151178418

0 days 12:20:00 0.906464459 0.632103274 0.361813857 0.490862293 0.151144151 0.721531432 0.285861516 0.152434467

0 days 12:30:00 0.917373042 0.639935987 0.365057572 0.496070404 0.152339352 0.72853832 0.288656887 0.153562219

0 days 12:40:00 0.928011641 0.647636322 0.368356697 0.501391652 0.15357631 0.735542916 0.291453382 0.154550205

0 days 12:50:00 0.93865239 0.65532261 0.37152009 0.506556195 0.154774998 0.74256405 0.294177303 0.155491987

0 days 13:00:00 0.949194558 0.662977785 0.374673308 0.511637079 0.156034682 0.749358808 0.296837639 0.156427904

0 days 13:10:00 0.959631088 0.670582073 0.37764728 0.516624039 0.157237526 0.756128557 0.299559335 0.157309875

0 days 13:20:00 0.970140162 0.678077414 0.380544781 0.521745651 0.158328107 0.762880837 0.302234479 0.158346155

0 days 13:30:00 0.980522925 0.685528014 0.383537189 0.526755757 0.15946379 0.769648345 0.304951306 0.159190755

0 days 13:40:00 0.989297866 0.691760763 0.385917343 0.530709723 0.160595208 0.775225467 0.307120194 0.16000363

0 days 13:50:00 0.999527985 0.699069254 0.388793134 0.535455749 0.161943627 0.781954474 0.309750095 0.160969107

0 days 14:00:00 1.009749692 0.706428191 0.391593168 0.540475483 0.163149067 0.788536901 0.312393667 0.16207563

0 days 14:10:00 1.019926736 0.713826259 0.394410472 0.545478561 0.164383922 0.795081644 0.314885797 0.162950452

0 days 14:20:00 1.030145581 0.72123693 0.397203585 0.55025723 0.165589474 0.801557056 0.317256088 0.163756294

0 days 14:30:00 1.040179858 0.728552081 0.399909236 0.555135217 0.166904727 0.808050783 0.31960935 0.164551841

0 days 14:40:00 1.050109104 0.735741568 0.402531108 0.55992848 0.168209381 0.814347404 0.321907191 0.165380851

0 days 14:50:00 1.06010561 0.742984239 0.405052946 0.564638469 0.169408098 0.820661414 0.324148315 0.166174646

0 days 15:00:00 1.069950345 0.750004015 0.407553275 0.569174062 0.170781215 0.826882232 0.326346609 0.167001252

0 days 15:10:00 1.078174531 0.755977784 0.409583242 0.572929138 0.17196112 0.831941751 0.328234809 0.16785437

0 days 15:20:00 1.087839988 0.762975429 0.412083449 0.577399664 0.173415127 0.838141569 0.330593412 0.168771326

0 days 15:30:00 1.097638947 0.770010011 0.414527186 0.581882871 0.174640161 0.84449411 0.332915712 0.169657663

0 days 15:40:00 1.10722692 0.776876463 0.416953472 0.586327572 0.175640044 0.850553419 0.335322946 0.170570037

0 days 15:50:00 1.116686786 0.783774385 0.419295833 0.590708656 0.176645283 0.856649646 0.337667206 0.171479207

0 days 16:00:00 1.12629773 0.790736065 0.421556894 0.594990807 0.177673538 0.862652308 0.340033245 0.172183438

0 days 16:10:00 1.13581985 0.797747255 0.423822438 0.599305529 0.178654383 0.868557107 0.342308925 0.17283749

0 days 16:20:00 1.145307107 0.804640363 0.426031764 0.603506732 0.179789619 0.874269675 0.344551489 0.173467286

0 days 16:30:00 1.154689193 0.811433335 0.428151938 0.607698329 0.180791981 0.879872581 0.346626142 0.174095777

0 days 16:40:00 1.163548166 0.818061236 0.430208341 0.611779041 0.181705212 0.885394472 0.348591815 0.174820912

0 days 16:50:00 1.17172728 0.823667726 0.432085983 0.615331722 0.182843194 0.890117374 0.350479617 0.175743825

0 days 17:00:00 1.180802763 0.830224865 0.434182431 0.619633862 0.183850173 0.895732214 0.352593393 0.17654802

0 days 17:10:00 1.190031411 0.836673413 0.436214466 0.623808054 0.184922736 0.901303055 0.35460951 0.17724734

0 days 17:20:00 1.199131154 0.843161039 0.438224265 0.628012008 0.185919766 0.906834843 0.356591266 0.177947426

0 days 17:30:00 1.207957995 0.849577888 0.440187381 0.631986108 0.18691205 0.912202636 0.358514702 0.178535216

0 days 17:40:00 1.216688541 0.855943071 0.442095122 0.636058373 0.187818015 0.917446365 0.360406319 0.179047756

0 days 17:50:00 1.225324296 0.862195923 0.443920817 0.640037045 0.18880737 0.92244081 0.362244116 0.179529015

0 days 18:00:00 1.233987944 0.868417329 0.4456985 0.644056198 0.18974968 0.927470627 0.364020262 0.180066973

0 days 18:10:00 1.242454617 0.874558712 0.447477787 0.647899552 0.190570018 0.932302563 0.365733953 0.18050565

0 days 18:20:00 1.249956676 0.879815357 0.448933702 0.651031129 0.191618613 0.936271646 0.36725472 0.181146379

0 days 18:30:00 1.258727666 0.885991637 0.450704407 0.654758085 0.192458586 0.940970516 0.369020959 0.181775775

0 days 18:40:00 1.26740279 0.892159505 0.45231537 0.658397906 0.193559657 0.945593587 0.370805521 0.182301535

0 days 18:50:00 1.276001501 0.898279235 0.453989206 0.66199052 0.194430963 0.950288637 0.372547218 0.182751971

0 days 19:00:00 1.284604336 0.904448393 0.455549107 0.66549915 0.195176005 0.954729963 0.374195948 0.183140873

0 days 19:10:00 1.292947931 0.910620082 0.457042519 0.668876295 0.195985737 0.9591747 0.375782067 0.183490922

0 days 19:20:00 1.301282539 0.916712002 0.458595284 0.672136723 0.196785636 0.96352894 0.377357502 0.183828419

0 days 19:30:00 1.309518351 0.922761853 0.460078391 0.675409273 0.197579448 0.967807405 0.378926215 0.184148215

0 days 19:40:00 1.317496948 0.928674664 0.461485818 0.678609676 0.198326903 0.971965996 0.380502032 0.184405864

0 days 19:50:00 1.324114222 0.933601219 0.462756205 0.681418141 0.19924403 0.975165495 0.381837745 0.184931129

0 days 20:00:00 1.331799353 0.939356326 0.464241291 0.684893408 0.200306391 0.978829745 0.383404507 0.185351518

0 days 20:10:00 1.339450247 0.945146892 0.465667374 0.688341607 0.201316557 0.982612651 0.38494013 0.185661518

0 days 20:20:00 1.347076134 0.95080796 0.467064748 0.691728015 0.202388479 0.986392702 0.386448325 0.18597798

0 days 20:30:00 1.354512703 0.956411562 0.468341026 0.695004033 0.203367446 0.990076241 0.387882784 0.186254006

0 days 20:40:00 1.361816534 0.96205907 0.469666696 0.698125047 0.204312424 0.993618591 0.389268587 0.186426232

0 days 20:50:00 1.36911637 0.967591598 0.470889494 0.701192628 0.20528612 0.997173905 0.39058486 0.186650947

0 days 21:00:00 1.376289986 0.973058384 0.472089454 0.704244659 0.20636254 1.0006284 0.391948175 0.186871542

0 days 21:10:00 1.383403133 0.978312862 0.473274667 0.707209604 0.207460922 1.003920701 0.393247135 0.187064028

0 days 21:20:00 1.389563796 0.982934765 0.474300771 0.709746333 0.208450442 1.006635087 0.394380601 0.187329185

0 days 21:30:00 1.397121099 0.988334922 0.475521298 0.712753888 0.209431685 1.009891594 0.395809694 0.187600334

0 days 21:40:00 1.404541697 0.993677843 0.476718293 0.71588998 0.210270699 1.013123653 0.397279535 0.187787582

0 days 21:50:00 1.411866442 0.999054546 0.477816769 0.718950552 0.211048495 1.016330037 0.398671557 0.187971725

0 days 22:00:00 1.419206851 1.004383235 0.478887622 0.722053616 0.211708728 1.019416926 0.400039025 0.188138146

0 days 22:10:00 1.426483903 1.009517073 0.479967669 0.725120908 0.212336776 1.022508622 0.401363681 0.18828475

0 days 22:20:00 1.433550251 1.014580017 0.480993009 0.728082085 0.212959134 1.025351912 0.402695953 0.18839809

0 days 22:30:00 1.440681895 1.019688452 0.481971946 0.731067494 0.213525007 1.028045927 0.404006464 0.188482987

0 days 22:40:00 1.447679481 1.024710793 0.482926323 0.733957067 0.214133242 1.030744873 0.405257438 0.188564809

0 days 22:50:00 1.453432022 1.028916588 0.483700355 0.736254372 0.21477704 1.032952059 0.406290807 0.188702135

0 days 23:00:00 1.459978202 1.03402378 0.484680142 0.7390315 0.215497798 1.035590041 0.407617354 0.188910678

0 days 23:10:00 1.466436283 1.038956971 0.48555996 0.741874795 0.216117237 1.038152328 0.408879671 0.189030874

0 days 23:20:00 1.473000649 1.04387162 0.486429979 0.744694464 0.216748002 1.040667393 0.410107352 0.189118178

0 days 23:30:00 1.479332193 1.048672653 0.487272482 0.747404843 0.217331594 1.043107588 0.411273239 0.189203582

0 days 23:40:00 1.485794325 1.053425079 0.488050381 0.750093939 0.217915572 1.045479271 0.412462783 0.189302528

0 days 23:50:00 1.492164024 1.0580937 0.488803673 0.752679897 0.218498771 1.047675919 0.413620047 0.189368793

1 days 00:00:00 1.498376201 1.0627448 0.489504307 0.75520073 0.219111793 1.049792293 0.414671865 0.189397451

1 days 00:10:00 1.504451577 1.067345186 0.490186694 0.757713839 0.219622443 1.051733489 0.415697855 0.189438087

1 days 00:20:00 1.50976592 1.071507404 0.490775706 0.759881063 0.220172341 1.05334635 0.416578535 0.189490016

1 days 00:30:00 1.515565757 1.075823852 0.491408972 0.762212109 0.220801825 1.055071164 0.417597712 0.189526327

1 days 00:40:00 1.521482775 1.080635781 0.492065671 0.764673749 0.221416862 1.056886934 0.418679315 0.189574674

1 days 00:50:00 1.527244076 1.085375087 0.492641457 0.76709652 0.222052432 1.058674239 0.419663333 0.189631582

1 days 01:00:00 1.53307452 1.089954333 0.493211294 0.769406242 0.222581875 1.060387911 0.420597419 0.189658654

1 days 01:10:00 1.53875294 1.094369221 0.493743293 0.771549493 0.223099542 1.061939703 0.421563996 0.189688731

1 days 01:20:00 1.544343574 1.09873882 0.494259627 0.773748666 0.223573718 1.063444422 0.422452011 0.189722102

1 days 01:30:00 1.549857686 1.102992194 0.494753388 0.775848302 0.224066626 1.064868437 0.423284591 0.189742154

1 days 01:40:00 1.555332361 1.10712696 0.4952198 0.777937223 0.224512712 1.066107205 0.42418014 0.18975202

1 days 01:50:00 1.560681097 1.111123944 0.495665907 0.779929228 0.225002867 1.067094081 0.424976068 0.189765634

1 days 02:00:00 1.565512235 1.11451213 0.496095169 0.781638144 0.225632708 1.067752805 0.42577839 0.189780045

1 days 02:10:00 1.571246646 1.118482238 0.496606275 0.783755406 0.226236961 1.068450468 0.42664801 0.189783034

1 days 02:20:00 1.576770271 1.12246262 0.4971149 0.785871616 0.226695165 1.06915374 0.427495369 0.189784634

1 days 02:30:00 1.58224261 1.126375166 0.497589596 0.78784869 0.22704382 1.069763661 0.428343815 0.189788869

1 days 02:40:00 1.587476946 1.13013966 0.498044661 0.78984422 0.227427815 1.070251595 0.429150295 0.189794787

1 days 02:50:00 1.592726284 1.133868335 0.498474421 0.791800486 0.227838064 1.070720968 0.429913097 NaN

1 days 03:00:00 1.59778423 1.137453955 0.498874607 0.793720389 0.228151316 1.071139578 0.430604993 0.189796288

1 days 03:10:00 1.602773417 1.140916289 0.499263441 0.79556759 0.228450423 1.071504053 0.431202494 0.189797847

1 days 03:20:00 1.60756041 1.144247011 0.499637813 0.797371542 0.228736161 1.071841438 0.431854321 0.189808434

1 days 03:30:00 1.611742782 1.14709307 0.499934848 0.798895112 0.229080429 1.072063207 0.432328113 0.189832301

1 days 03:40:00 1.61676696 1.150405473 0.500278456 0.800679528 0.229409936 1.072282401 0.432872372 0.18984378

1 days 03:50:00 1.621712898 1.153707742 0.500571749 0.802479002 0.22972227 1.072482057 0.433415821 0.189852136

1 days 04:00:00 1.626516735 1.156923019 0.500841808 0.804265321 0.230006598 1.072628723 0.433932448 0.189874712

1 days 04:10:00 1.631213597 1.160018287 0.501129945 0.805901391 0.230337633 1.072754643 0.434428538 0.189884541

1 days 04:20:00 1.63581392 1.162965295 0.501380731 0.807445602 0.23057072 1.072827812 0.434905487 0.189894023

1 days 04:30:00 1.640256852 1.165948643 0.501612773 0.808925495 0.23081483 1.072882588 0.435330025 0.189907485

1 days 04:40:00 1.644656045 1.168827611 0.501777603 0.810386259 0.23100369 1.07289968 0.435757011 0.189915148

1 days 04:50:00 1.648889678 1.171639396 0.501955268 0.811804891 0.231183178 1.072921061 0.436156515 0.189927267

1 days 05:00:00 1.652552832 1.173940345 0.502079341 0.812915668 0.231535876 1.07293352 0.436495312 0.189941208

1 days 05:10:00 1.657104691 1.17662582 0.502176934 0.814146098 0.231699381 1.072951411 0.436889429 0.18994485

1 days 05:20:00 1.661447787 1.179230341 0.502275388 0.815277874 0.231822471 1.072961211 0.437269114 0.189946553

1 days 05:30:00 1.665667948 1.181768329 0.502355805 0.81634392 0.231992631 1.072974318 0.437580487 0.189953869

1 days 05:40:00 1.669815128 1.184256703 0.50243684 0.817469965 0.232043037 1.072974384 0.437906816 0.189956879

1 days 05:50:00 1.673845286 1.186578497 0.502510276 0.818413097 0.232080403 1.072982982 0.438231559 0.189960422

1 days 06:00:00 1.677830994 1.188857933 0.50257606 0.819290322 0.232102489 1.072998233 0.43856515 0.189964726

1 days 06:10:00 1.681690641 1.191015362 0.502623261 0.820157663 0.232126761 1.073037195 0.438848978 NaN

1 days 06:20:00 1.685457303 1.193146124 0.502672693 0.820986077 0.232141163 1.073081635 0.439082583 0.189965517

1 days 06:30:00 1.688606396 1.194881754 0.502712817 0.821521176 0.232284575 1.073106972 0.439280225 0.189977433

1 days 06:40:00 1.692625919 1.197190937 0.502752701 0.822127913 0.23233526 1.073128125 0.439569205 0.189989919

1 days 06:50:00 1.696609317 1.199499192 0.502790106 0.82272332 0.232364052 1.073147617 0.439821228 0.189999706

1 days 07:00:00 1.70053252 1.201708776 0.502827039 0.823203839 0.232424584 1.0731586 0.440072231 0.190005432

1 days 07:10:00 1.704279488 1.203862031 0.502863145 0.823595966 0.232441431 1.073168214 0.440303451 0.190008874

1 days 07:20:00 1.707970989 1.20598111 0.502887078 0.823931733 0.232450597 1.0731689 0.440507076 0.190013867

1 days 07:30:00 1.711545844 1.207964359 0.502904826 0.824195714 0.232458593 NaN 0.440706825 0.190014431

1 days 07:40:00 1.714926482 1.20993427 0.502924049 0.824421387 0.232469654 NaN 0.440889485 0.190022662

1 days 07:50:00 1.718247072 1.211819651 0.502944246 0.824587026 0.232476683 NaN 0.441054987 0.190025263

1 days 08:00:00 1.720973769 1.213463118 0.502953172 0.8247161 0.232548186 1.07317142 0.441199611 0.190028343

1 days 08:10:00 1.72434987 1.215167609 0.502956239 0.824810347 0.232575161 1.073184976 0.441427753 0.190028974

1 days 08:20:00 1.727640343 1.216972233 0.502959365 0.824903552 0.232594123 1.073228553 0.441621105 0.190029203

1 days 08:30:00 1.730806634 1.21871022 0.502960949 0.824987205 0.232609241 1.073280368 0.441806631 0.19003251

1 days 08:40:00 1.733838572 1.220382655 0.502963427 0.825042248 0.232617987 1.073303447 0.441987403 0.190032849

1 days 08:50:00 1.736921112 1.221983125 0.502964112 0.825089685 0.232630465 1.073310485 0.442159994 NaN

1 days 09:00:00 1.739794476 1.223437087 0.502967608 0.82512383 0.232640349 1.07331748 0.44232093 NaN

1 days 09:10:00 1.742584507 1.224848423 0.502975196 0.82514926 0.232650723 1.073319168 0.442445763 NaN

1 days 09:20:00 1.745325452 1.226196925 0.502980201 0.825158636 0.232658551 NaN 0.442582144 NaN

1 days 09:30:00 1.747908948 1.227505212 0.502981931 0.825165994 0.232700772 NaN 0.442688796 0.190036125

1 days 09:40:00 1.750395565 1.228624139 0.502985987 0.825172805 0.232719681 1.073375425 0.442827373 0.19003649

1 days 09:50:00 1.753212534 1.229987543 0.502992811 0.825187051 0.232730172 1.073434604 0.44298106 0.190036822

1 days 10:00:00 1.755921742 1.231265784 0.502995935 0.825200929 0.2327378 1.073455533 0.443136672 0.190039407

1 days 10:10:00 1.758575984 1.232466415 0.502999484 0.825214903 0.232744114 1.073476447 0.443266802 NaN

1 days 10:20:00 1.761088111 1.2335832 0.503000801 0.82522996 0.23275341 1.073480907 0.443394374 NaN

1 days 10:30:00 1.763579441 1.23466415 0.503002207 0.825242185 0.232762905 NaN 0.443521263 NaN

1 days 10:40:00 1.765932723 1.235668877 NaN 0.825256038 0.232784121 NaN 0.443623825 NaN

1 days 10:50:00 1.768204667 1.23661594 NaN 0.825272502 0.232789529 NaN 0.443721573 NaN

1 days 11:00:00 1.770345195 1.237501885 NaN 0.825287903 0.232831663 NaN 0.443791432 0.190044605

1 days 11:10:00 1.772051303 1.238381182 0.503003154 0.825299096 0.23284351 1.073508359 0.443843756 0.190052898

1 days 11:20:00 1.773946415 1.239486303 0.503003899 0.825308333 0.232853627 1.07354651 0.443928878 0.190067111

1 days 11:30:00 1.775821356 1.240514341 0.503006442 0.825319898 0.232861097 1.073561249 0.443990198 0.190071347

1 days 11:40:00 1.777676833 1.241432411 0.503008349 0.825326902 0.232868158 1.073569073 0.444058395 0.190078668

1 days 11:50:00 1.779478112 1.242272346 0.503009113 0.825338919 0.232876303 1.073574722 0.44410408 0.19007936

1 days 12:00:00 1.781105017 1.243141515 0.503009832 0.825348442 0.232892212 NaN 0.444145731 NaN

1 days 12:10:00 1.782709372 1.243891712 NaN 0.825367391 0.232897812 NaN 0.444223188 0.190080518

1 days 12:20:00 1.784194769 1.244611299 NaN 0.825384093 0.23290162 NaN 0.444268703 NaN

1 days 12:30:00 1.785657692 1.245284863 NaN 0.825396579 0.232921265 NaN 0.444302863 0.190084419

1 days 12:40:00 1.786934673 1.245785245 0.503010006 0.825402559 0.23293153 1.073584967 0.444334119 0.190091755

1 days 12:50:00 1.78846406 1.24649146 0.50301101 0.82541021 0.232935812 1.073620803 0.444362963 NaN

1 days 13:00:00 1.790031346 1.247142224 0.503013921 0.825411211 0.232939263 1.0736215 0.444390799 NaN

1 days 13:10:00 1.791501704 1.247703694 0.503014785 0.825411257 0.232942787 NaN 0.444413127 NaN

1 days 13:20:00 1.792927586 1.248260447 0.503016329 0.825411707 0.232948515 1.073622809 0.444438494 NaN

1 days 13:30:00 1.794320318 1.248756869 0.503017543 0.825412426 0.232950657 1.073625543 0.44445883 NaN

1 days 13:40:00 1.795656024 1.249210482 NaN 0.825419871 0.232953152 NaN 0.44447201 NaN

1 days 13:50:00 1.796890655 1.249605965 NaN 0.825430523 0.232954632 NaN 0.444480702 NaN

1 days 14:00:00 1.798012911 1.249959081 NaN 0.825435268 0.232959939 NaN 0.444492518 NaN

1 days 14:10:00 1.79887957 1.250234197 0.503019678 0.825441116 0.232963671 1.073628556 0.444501126 0.190092377

1 days 14:20:00 1.799883929 1.250533777 0.503022536 0.825445587 0.232966339 1.073631343 0.444515227 0.190093298

1 days 14:30:00 1.800828426 1.250780299 0.503024116 0.825458551 0.232968201 NaN 0.444526797 NaN

1 days 14:40:00 1.801776211 1.251010685 0.503026481 0.825471572 0.232971208 NaN 0.444538869 NaN

1 days 14:50:00 1.802557236 1.25121692 0.503028772 0.825477606 0.232973683 NaN 0.444553865 NaN

1 days 15:00:00 1.803252334 1.251383298 NaN NaN 0.232975989 NaN 0.444573033 NaN

1 days 15:10:00 1.803865353 1.251522891 0.503028778 NaN 0.232976521 NaN 0.444584232 NaN

1 days 15:20:00 1.804425012 1.251650246 NaN NaN 0.232977726 NaN 0.444596084 NaN

1 days 15:30:00 1.804998226 1.251764312 NaN NaN 0.232980839 NaN 0.444606236 NaN

1 days 15:40:00 1.805441966 1.251863392 0.503029448 0.825478301 0.232986328 1.073633809 0.444608955 0.190108195

1 days 15:50:00 1.806003547 1.251985702 0.503030165 0.825501999 0.232989047 1.073646946 0.444613806 0.190108337

1 days 16:00:00 1.806551084 1.252095479 0.503033401 0.825536727 0.232990315 NaN 0.44461649 0.190108623

1 days 16:10:00 1.807072294 1.252203536 0.503036228 0.82556534 0.232991179 NaN 0.444617695 NaN

1 days 16:20:00 1.807608274 1.252298123 0.503039991 0.825577451 0.23299207 NaN 0.444619747 NaN

1 days 16:30:00 1.808103191 1.252402988 0.503041149 0.82558462 0.232993235 NaN 0.44462108 NaN

1 days 16:40:00 1.808618591 1.252464904 NaN NaN 0.232994627 NaN 0.44462253 NaN

1 days 16:50:00 1.80905677 1.252530448 NaN NaN 0.232995519 NaN 0.444623075 NaN

1 days 17:00:00 1.809483225 1.252577183 0.503041197 NaN 0.232999036 NaN 0.444627091 0.190109023

1 days 17:10:00 1.809836373 1.252627364 NaN NaN 0.233021251 NaN 0.444627645 0.190109924

1 days 17:20:00 1.810088878 1.252676851 0.503042168 0.82558945 0.233022393 1.073650997 0.444628622 0.190110337

1 days 17:30:00 1.810366006 1.252735306 0.503044089 0.825600574 0.233023163 NaN 0.444628724 NaN

1 days 17:40:00 1.81062994 1.252774234 0.503052925 NaN 0.233028351 NaN 0.444629435 NaN

1 days 17:50:00 1.810875747 1.252813757 NaN NaN 0.233031253 NaN 0.444632989 NaN

1 days 18:00:00 1.81112196 1.252838378 NaN NaN 0.233032783 NaN 0.444634615 NaN

1 days 18:10:00 1.811359425 1.252859906 NaN NaN 0.233034837 NaN 0.44463569 NaN

1 days 18:20:00 1.811561976 1.252889094 NaN NaN 0.233035247 NaN 0.444637657 0.190112076

1 days 18:30:00 1.811747837 1.252908818 NaN NaN 0.233036007 NaN 0.444639986 NaN

1 days 18:40:00 1.811908231 1.252926915 NaN NaN 0.233038291 NaN 0.444642365 NaN

1 days 18:50:00 1.812133958 1.25294681 0.503053029 0.825609543 0.233038641 1.073653862 0.444645571 NaN

1 days 19:00:00 1.812366098 1.252957713 0.50305372 0.825623589 NaN NaN 0.444646736 NaN

1 days 19:10:00 1.812593937 1.25297119 0.50305484 0.825623913 0.23303917 NaN NaN NaN

1 days 19:20:00 1.812792394 1.252977251 NaN NaN 0.233039333 NaN 0.444648106 NaN

1 days 19:30:00 1.812969664 1.252977846 NaN NaN 0.233039885 NaN NaN NaN

1 days 19:40:00 1.813136229 1.252978679 NaN NaN 0.233039967 NaN 0.444648316 NaN

1 days 19:50:00 1.813263075 1.252979127 NaN NaN 0.23304037 NaN 0.444648896 NaN

1 days 20:00:00 1.813367545 NaN NaN NaN 0.233041663 NaN 0.444649664 NaN

1 days 20:10:00 1.813463728 NaN NaN NaN 0.23304238 NaN NaN NaN

1 days 20:20:00 1.813540189 1.252980177 NaN 0.825632779 0.233042485 NaN 0.444650034 NaN

1 days 20:30:00 1.813628912 1.252981652 NaN 0.825647452 0.233042597 NaN NaN NaN

1 days 20:40:00 1.813716689 1.252986941 NaN 0.825655404 NaN NaN 0.444650109 NaN

1 days 20:50:00 1.813764246 1.252989499 NaN 0.825657404 0.233042881 NaN NaN NaN

1 days 21:00:00 1.813817475 1.252999745 NaN NaN NaN NaN NaN NaN

1 days 21:10:00 1.81386961 1.253006888 NaN NaN 0.233043077 NaN NaN NaN

1 days 21:20:00 1.813920842 1.253014477 NaN NaN 0.233044739 NaN NaN NaN

1 days 21:30:00 1.813969715 1.253018702 NaN NaN 0.233045086 NaN NaN NaN

1 days 21:40:00 1.814012372 1.253019838 NaN NaN 0.233045588 NaN NaN 0.190112389

1 days 21:50:00 1.814038764 1.253029135 NaN 0.825660502 NaN NaN 0.444650802 NaN

1 days 22:00:00 1.814072695 1.253042257 NaN 0.825675522 NaN NaN 0.444652058 NaN

1 days 22:10:00 1.8141247 1.253063183 NaN 0.825679338 NaN NaN 0.444655557 NaN

1 days 22:20:00 1.814153958 1.253079742 NaN NaN NaN NaN 0.444657268 NaN

1 days 22:30:00 1.814173702 1.253090586 NaN NaN NaN NaN 0.444658479 NaN

1 days 22:40:00 1.814186285 1.253097801 NaN NaN NaN NaN 0.444661236 NaN

1 days 22:50:00 1.814191576 1.25309886 NaN NaN NaN NaN 0.444665509 NaN

1 days 23:00:00 1.814207947 NaN NaN NaN NaN NaN 0.444668536 NaN

1 days 23:10:00 1.814219103 NaN NaN NaN NaN NaN 0.444668957 0.190112771

1 days 23:20:00 1.814232657 1.253099487 NaN 0.825682185 NaN NaN 0.444669252 NaN

1 days 23:30:00 1.814253226 1.253106086 NaN 0.82569457 NaN NaN 0.444669627 NaN

1 days 23:40:00 1.814266085 1.253112951 NaN 0.825695249 NaN NaN NaN NaN

1 days 23:50:00 1.814273701 1.253113279 NaN NaN NaN NaN NaN NaN
